# Integrative transcriptome analysis discloses the molecular basis of a heterogeneous fungal phytopathogen complex, *Rhizoctonia solani* AG-1 subgroups

**DOI:** 10.1038/s41598-019-55734-2

**Published:** 2019-12-23

**Authors:** Naoki Yamamoto, Yanran Wang, Runmao Lin, Yueyang Liang, Yao Liu, Jun Zhu, Lingxia Wang, Shiquan Wang, Huainian Liu, Qiming Deng, Shuangcheng Li, Ping Li, Aiping Zheng

**Affiliations:** 1 Rice Research Institute, Sichuan Agricultural University, Chengdu, 611130 China; 2 Key Laboratory of Sichuan Crop Major Diseases, Sichuan Agricultural University, Chengdu, 611130 China; 3 Key Laboratory of Southwest Crop Gene Resource and Genetic Improvement of Ministry of Education, Sichuan Agricultural University, Ya’an, 625014 China; 4 Present Address: Institute of Vegetables and Flowers, Chinese Academy of Agricultural Sciences, Beijing, 100081 China

**Keywords:** Transcriptomics, Pathogens

## Abstract

*Rhizoctonia solani* is a fungal species complex that causes necrotrophic crop diseases. It comprises several anastomosis groups, some of which include intra-subgroups, such as AG-1 IA and AG-1 IB, exhibiting varying pathogenicity. Owing to its heterozygous and multinucleate features, genomic analyses of *R. solani* are still challenging, and understanding of its genetic diversity and genic components is limited. In this study, in order to elucidate the molecular basis of this phytopathogen complex, an integrated transcriptome analysis was undertaken for three subgroups of AG-1, i.e. AG-1 IA, AG-1 IB, and AG-1 IC. Sequence variations suggested substantial evolutionary distances within AG-1. Transcript simple sequence repeats showed comparable characteristics among AG-1, but contained polymorphic sites. Intra-subgroup polymorphisms suggested varying genic heterozygosity within AG-1, suggesting their independent evolutionary trajectory. Sequences of pathogenic factors, phytotoxin biosynthesis pathway enzymes, secreted lignocellulosic enzymes, secreted reactive oxygen species detoxification enzymes, apoplastic/cytoplasmic effector candidates, were conserved among those subgroups. *d*_N_/*d*_S_ ratios of a secretome subset suggested core secreted proteins in AG-1 and distinct evolution of Cys-rich small secreted proteins after differentiation of AG-1 subgroups. Identification of likely pathogenic factors including allergen protein homologues, oxidative phosphorylation and ethylene biosynthesis pathways, and diversification of polysaccharide monooxygenases provides molecular insight into key genomic components that play a role in *R. solani* pathogenesis.

## Introduction

*Rhizoctonia solani* is a soil-borne Basidiomycete fungus that causes crop diseases such as ‘sheath blight’, ‘web blight’, or ‘leaf blight’^1^. *R. solani* can infect various plant species including cereal crops, beans, pasture crops, oil crops, and vegetable crops to produce necrotic symptoms^1–3^. This fungus is a species complex with relatively diverse morphology, physiology, host specificity, and pathogenicity^4^. At present, *R. solani* isolates are classified into at least 14 anastomosis groups (AGs): AG-1 to AG-13 and a bridging isolate AG-BI, which differ from each other in terms of their anastomosis behavior^4^. AG-1 comprises intraspecific subgroups including AG-1 IA, AG-1 IB, and AG-1 IC, which exhibit varying pathogenicities^5^. AG-1 IA is responsible for rice sheath blight, which causes yield loss ranging from 20 to 70% depending on the environmental conditions^6^, while AG-1 IB and AG-1 IC are less virulent to rice^5^. Sheath blight is the second most serious disease affecting rice production, especially in East and Southeast Asia and the southern United States. To develop eco-friendly control strategies for this disease, an improved understanding of *R. solani* and its pathogenic mechanisms is necessary.

Recent studies of *R. solani* have emphasized the importance of the role of bioactive molecules in infection processes as well as physical attacks of hyphae to plant tissues. Brooks *et al*.^7^ and Hu *et al*.^8^ reported that phytotoxins, i.e., phenylacetic acid (PAA) and 3-methylthioproprionic acid (MTPA), are likely responsible for producing disease symptoms caused by *R. solani*. In addition, an array of enzymes for degrading plant cell walls are encoded by this fungal genome, and the corresponding genes are expressed during disease pathogenesis^9–11^. Furthermore, secreted proteins that appear not to encode enzymes for producing necrosis were identified in this fungal species^10,12^. Such ‘effector’ proteins would be able to elicit necrosis by triggering programmed cell death (PCD)^13^. The significance of this key finding is emphasized by the association between PCD and *R. solani* infection^14^.

Comparative sequence analyses should yield novel insights into closely related *R. solani* strains that exhibit different phenotypes. However, owing to the multinucleate nature of *R. solani*, genetic analyses of this species require substantial efforts using large-scale sequencing data. Several draft genome sequences of *R. solani* strains, e.g. the national standard isolate of AG-1 IA (strain AG1 IA), AG-1 IB 7/3/14 from lettuce, AG-2-2 IIIB from sugar beet, AG-3 from potato, and AG-8 from lupin, have been constructed^15,16^. These genome sequences are fragmented, and intraspecific genomic diversity of *R. solani* complicates comparison of genetic information. Therefore, *de novo* transcriptome sequencing is useful for analyzing the genetic diversity and genic components of *R. solani*. At present, RNA sequencing (RNA-Seq) data of the AG-1 IA strain after inoculation onto rice and expressed sequence tags of the AG-1 IB isolate 7/3/14 are available^10,11^. Host immunity affects regulation of gene expression in *R. solani*; therefore, construction of a basal transcriptome profile (without inoculation on host plants) of the AG-1 IA strain should yield novel insights into the genes involved in the pathogenesis of rice sheath blight. Moreover, mycelial transcriptome sequences of AG-1 IB and AG-1 IC represent useful resources for investigating the shared and differing genomic/physiological/pathogenic characteristics within the AG-1 subgroup.

In this study, we conducted deep mRNA sequencing in cultured mycelia of AG1 IA, with one isolate each for AG-1 IB and AG-1 IC. Transcriptome sequences were characterized to examine (1) sequence diversity among the AG-1 subgroups and multinucleate DNAs, (2) gene repertoire of the transcriptomes and the secretomes, and (3) candidate genes underlying the pathogenicity of *R. solani*. For improved interpretation, the previously generated genome and transcriptome sequencing data in AG1 IA were utilized. Our integrated analysis yields novel insights into the complex pathogenic mechanisms of *R. solani* and expands knowledge on genic variability, detailed genic repertoire, and functional molecules in AG-1 and fungal blight diseases. This study is the first comprehensive report of the characteristics of genic sequences in multi-subgroups of an *R. solani* AG.

## Results and Discussion

### Transcriptome sequencing and *de novo* assembly

Paired-end short reads of mycelial transcriptomes were obtained by Illumina high-throughput sequencing. Millions of paired-end reads were generated from poly (A)^+^ RNA fractions of mycelia in AG-1 IA, AG-1 IB, and AG-1 IC, respectively (Table 1). Our preliminary trial of batch assembly of these sequencing data was unsuccessful; therefore, the sequencing data were separately assembled into contigs (22,845 transcriptome contigs (IA-TCs) for AG-1 IA; 21,905 transcriptome contigs for AG-1 IB (IB-TCs); and 19,719 transcriptome contigs for AG-1 IC (IC-TCs)) using Trinity^17^. The results obtained using Trinity were superior in terms of revealing conserved genes among fungi and basidiomycetes to those obtained using Velvet/Oases^18^. The Illumina reads exhibited good re-mapping rates (96.9 to 98.7%) in all AG-1 subgroups. The assembly statistics, N50 length, and data showing the existence of conserved orthologous genes in Basidiomycota and fungi were similar among the subgroups (Table 1). GC contents of the contigs were 50.8, 51.4, and 51.3% in IA-TCs, IB-TCs, and IC-TCs, respectively. These were similar to that of the transcript contigs of AG-1 IB 7/3/14 (50.3%) and slightly higher than the GC content of the AG-1 IA reference genome (47.6%)^10,11^. A total of 21,627 AG-1 IA contigs (94.7%) could be mapped upon the AG-1 IA genome sequence using BLAT^19^. A total of 19,640 of IB-TCs (89.7%) could be mapped onto all the coding sequences of AG-1 IB 7/3/14 by BLAT with the E-value of 1e-5; the sequence identities ranged from 64.2% to 100%, with an average of 98.4%; 1,864 IB-TCs exhibited 100% sequence identity with their counterparts in AG-1 IB 7/3/14. These results indicate the accuracy of the present assemblies.

**Table 1:** Data statistics of the transcriptome sequencing in AG 1 subgroups.

	IA-TCs	IB-TCs	IC-TCs	IA-STCs
Number of paired-end reads	6,367,931	6,038,800	5,104,151	35,555,860
Number of contigs	22,845	21,905	19,719	43,553
Total nucleotides in contigs (bp)	19,657,497	20,550,584	16,395,799	63,798,492
N50 length of contigs (bp)	1,336	1,397	1,199	2,424
Average length of contigs (bp)	861	938	832	1,465
GC content (%)	50.8	51.4	51.3	50.3
Minimum length of contigs (bp)	201	201	201	201
Maximum length of contigs (bp)	12,071	12,107	9,686	13,306
BUSCO (fungi)	273/290	283/290	282/290	289/290
BUSCO (basidiomycetes)	1,121/1,335	1,215/1,335	1,205/1,335	1,320/1,335

*Number of present genes/all conserved common genes tested by BUSCO.

Pairwise comparisons of the transcriptome contigs indicated significant nucleotide diversity within the AG-1 subgroups. Specifically, BLASTN^20^ searches with the E-value of 0.01 revealed nucleotide sequence identities of 85.3 to 87.1% on average between probable orthologous transcripts; however, sequence identities varied depending on genes. For example, IA-TC and IB-TC transcripts of housekeeping genes, i.e. *glyceraldehyde-3-phosphate dehydrogenase* and *alcohol dehydrogenase*, showed almost absolute identity (99.8 and 100%, respectively), while transcripts encoding regulatory genes, *zinc finger protein* and *bZIP-type transcription factor*, exhibited lower sequence homologies (84.5 and 82.8%, respectively). Only approximately the halves of the contigs showed significant sequence similarities between different subgroups; actual sequence conservation would be lower than that observed. According to our preliminary estimation of divergence time based on six of 50 housekeeping Clusters of Orthologous Groups (COGs)^21^, AG-1 IA and AG-1 IB diverged around 46.2 million years ago (MYA), and the lineage of these two subgroups diverged from AG-1 IC around 76.1 MYA. Although these values require evaluation using whole-genome sequences, this result is consistent with those of previous reports that describe divergent DNA sequences in AG-1^22,23^.

### Super-transcriptome contigs in AG-1 IA for rice sheath blight

To capture transcripts that are inducible by inoculation and/or infection onto plant hosts, we conducted batch transcriptome assembly of AG-1 IA by integrating previously generated RNA-Seq data in mycelia after inoculation onto rice^10^; 43,553 super-transcriptome contigs (IA-STCs) were constructed (Table 1). The IA-STCs showed good re-mapping rates (94.0 to 98.6%) in the AG 1-IA RNA-Seq profiles. As indicated by the assembly statistics of IA-STCs (Table 1), this contig set has higher coverage in the transcriptome because of the greater number of total nucleotides and common genes in fungi and Basidiomycetes than in IA-TCs. Notably, 10,168 IA-STCs were uniquely detected after inoculation only. The expression of most of these IA-STCs was detected 48 hours or later after inoculation: the number of IA-STCs increased to 8,713 at 48 hours and 9,666 at 72 hours after inoculation (Table S1). These transcripts may be involved in biological processes underlying host tissue necrosis during *R. solani* infection.

Unexpectedly, 10,775 IA-STCs (24.8%) were not mapped to the AG-1 IA genome assembly. Among these IA-STCs, approximately 3,000 had almost identical sequences to those of rice. The remaining IA-STCs may have been derived from other microorganisms growing along with the inoculated AG-1 IA isolate. Another possibility is that the reference genome did not cover some genic regions. To test the latter possibility, we predicted the genome size of AG-1 IA by a *k*-mer analysis using the two sets of genomic DNA sequencing data with the 173 bp and 5.6 kb inserts^10^ using the method of Yamamoto *et al*.^24^.The estimated genome size ranged from 41.4 to 49.3 Mb at the *k*-mer length of 17. These values are 11.2 to 33.4% larger than the total length of the genome assembly. Hence, we assumed that some of the 10,775 IA-STCs were transcribed from the AG-1 IA genome. Contraction of the IA-STCs using EvidentialGene^25^ resulted in the retention of 16,334 contigs as the representative set of IA-STCs, implying an approximately 1.5-fold greater number of genes in *R. solani* AG-1 IA than the previously predicted number of 10,489 on the draft genome sequence^10^. The AG-1 IB 7/3/14 genome and annotation statistics showed that more than 40 Mb of the genome and 15 thousands genes were possibly encoded in *R. solani* AG-1^15^. Further analyses of the AG-1 IA genome and transcriptome may lead to the identification of uncharacterized genes.

### Simple sequence repeats in the mycelial transcriptomes

**Figure 1:**
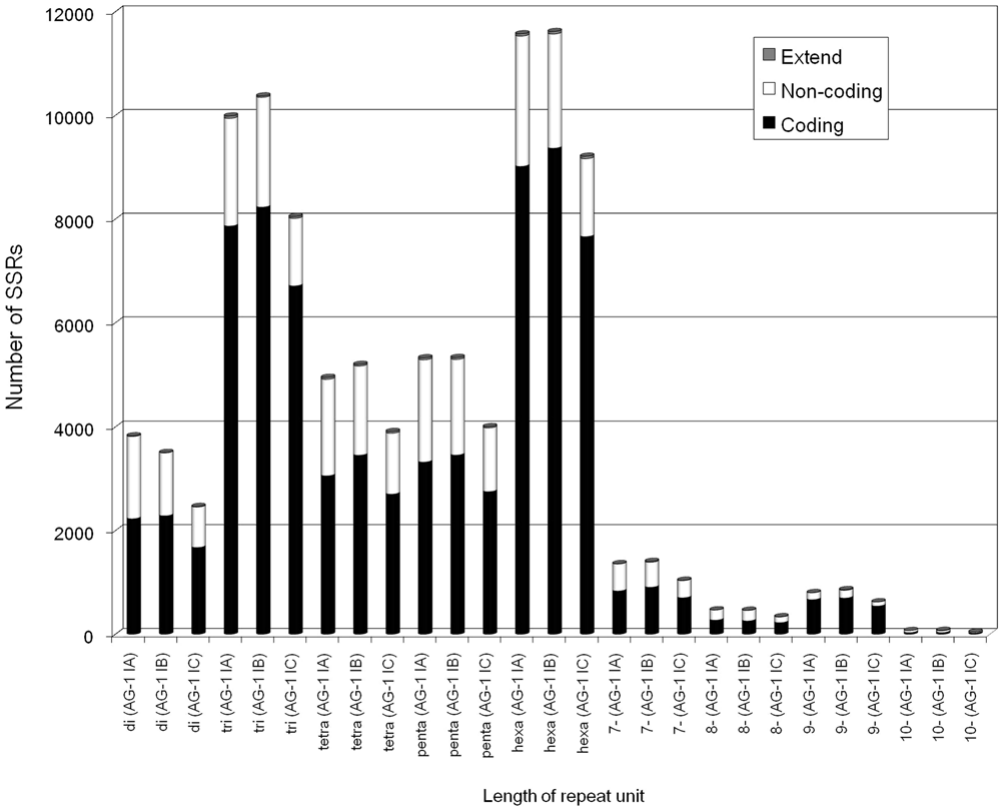
Distribution of SSRs on the mycelial transcriptome contigs. The SSRs with 2–10 bases of units were shown. Extend: SSRs extending between non-coding and coding sequences, Non-coding: SSRs in non-coding sequence, Coding: SSRs in coding sequence.

Profiling of simple sequence repeats (SSRs) provides an alternative index for global sequence similarity in the transcriptomes because of the high mutation rates of repeat sequences. Genic SSRs are likely to be under distinct selective pressure^26^ and may represent evolutionary adaptation at gene level. In addition, studies have demonstrated that genic SSRs show transferability across genetically close species^27,28^. Via a computational search, we detected 38,603 (2.0 SSRs/kb), 39,102 (1.9 SSRs/kb) and 29,863 (1.8 SSRs/kb) SSRs on IA-TCs, IB-TCs, and IC-TCs, respectively. Most IA-TCs SSRs were validated by comparison with the genomic sequences (data not shown). The majority of these SSRs comprised up to six nucleotide units (Fig. 1), which is a typical feature of genic SSRs^26^. Transcript SSRs of tri- and hexa-nucleotide units were more frequent owing to larger numbers of coding SSRs, indicating that SSRs that did not cause frame shifts were evolutionarily retained on the coding sequence of the AG-1 subgroups, and that transcripts with such SSRs would be functionally important.

Frequencies of allelic types of repeat units differed only slightly among the AG-1 subgroups (Fig. S1A), indicating conserved sequence characteristics in the transcriptomes. The most frequent unit was ‘AAG’, followed by ‘AG’, ‘AGC’, ‘ACC’, and ‘ACG’, which occur frequently in fungi^26,29^. Similar but different frequencies of coding SSRs represent conserved sequence characteristics in the transcriptomes among the AG-1 subgroups (Fig. S1B), suggesting the occurrence of polymorphic coding SSRs in the subgroups. In fact, manual survey for coding SSRs with tri-, hexa-, 9-, and 12-nucleotide repeat units revealed several SSR marker candidates that were capable of differentiating the AG-1 subgroups (Fig. S2). Surrounding transcript sequences of the SSRs were highly conserved in AG-1; therefore, such transcriptome SSRs provide an alternative genotyping strategy for *R. solani*.

### Multinucleate transcript polymorphisms

Natural multinucleate cells have been observed in *R. solani* AG-1 strains^5,10^. Genetically diverse nuclei support phenotypic plasticity in mycelia and contribute to fungal virulence^30^. Our previous microscopic analysis revealed that the AG-1 IA reference strain possesses eight to 10 nuclei per cell^10^. Grosch *et al*.^31^ observed that AG-1 IB isolates had six nuclei per cell on average. Consistent with this report, present microscopic observations in AG-1 IC indicated around six nuclei per cell (data not shown). To investigate heterozygotic status in the AG-1 strains, we mined probable single nucleotide polymorphisms (SNPs), multi-nucleotide polymorphisms (MNPs), and short insertions and deletions (InDels) on the transcriptome contigs (Table S2). The frequencies of the various polymorphisms differed: AG-1 IB was the most frequent and AG-1 IC was the least frequent. A referential data for AG-1 IA genome supported the reliability of these results, implying intra-genetic heterozygosity at DNA level. The frequencies of multinucleate SNP sites in the transcriptomes were much lower than that of the AG-8 genome (14.5 coding SNPs/kbp)^32^ but similar to or less than those of fungi such as *Lentinula edodes* (4.6 SNPs/kbp)^33^ and *Blumeria graminis* (1.0 SNP/kbp)^34^. Allelic types showed that substitutions between purine or pyrimidine bases were predominant in all subgroups (Table S3), implying that these nucleotide variants were derived from mutations during evolution.

### Transcript repertoire

The transcriptome contigs were annotated with KOGs^35^, Swiss-Prot^36^, Pfam protein domains^37^, KEGG metabolic pathways^38^, PHI-base^39^ and CAZymes (carbohydrate-active enzymes)^40^. In addition to the longest open reading frames, which most probably encode proteins, alternative frames can be biologically significant eukaryotes^41,42^. Therefore, all the deduced protein sequences with no fewer than 50 amino acid residues were analyzed. In our coding sequence prediction, 4.6 potential coding sequences per transcriptome contig were found on average. Sequence searches of the predicted protein sequences resulted in the assignment of any type of annotation to 12,696 (55.6%) IA-TCs, 12,368 (56.5%) IB-TCs, 11,310 (57.4%) IC-TCs, and 27,961 (64.2%) IA-STCs (Table S4). KOG categorization resulted in similar distributions among the four transcriptome contig sets (Fig. S3), suggesting conservation of the fundamental gene set. Sequence searches against the reference fungal proteomes indicated considerable numbers of homologues in *R. solani*, *Laccaria bicolor*, and *Coprinopsis cinerea*, which are categorized as Basidiomycota (Table S5). These findings were largely consistent with the taxonomic relationship. The following unique gene sequences for the five necrotrophs were found: *endoglucanase A* [UniProt accession: O08342], *endoglucanase B* [P23550], and *cytochrome P450* [Pfam accession: PF00067.21] (Table S6). The functions of the other listed contigs listed could not be predicted; however, it is considered that contigs may play specific roles in necrotrophs.

**Figure 2:**
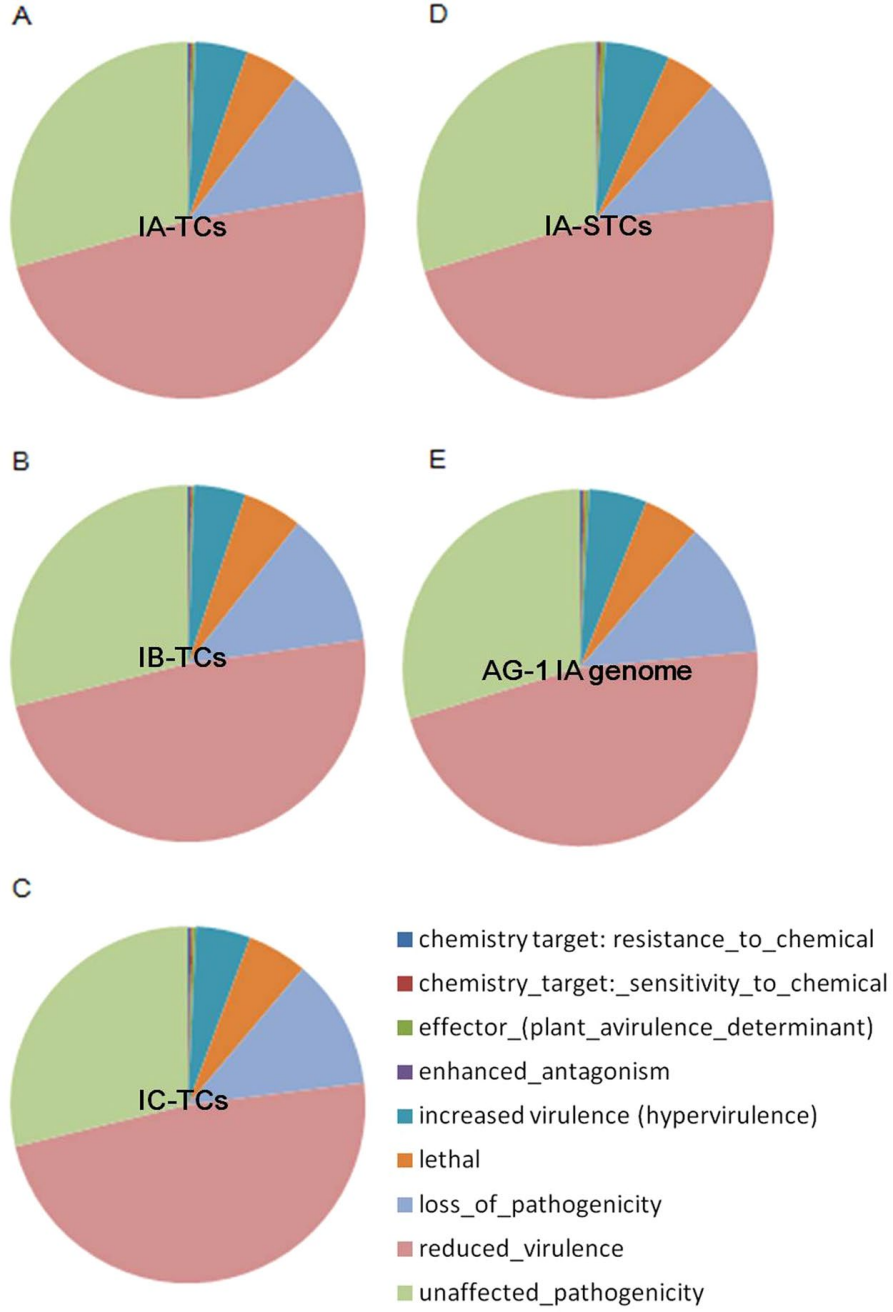
Distribution of PHI-base accessions of which sequences showed homologies with predicted proteins in the three AG-1 strains. Numerals represent numbers of PHI-base accessions. (**A**) IA-TCs, (**B**) IB-TCs, (**C**) IC-TCs, (**D**) IA-STCs, and (**E**) the AG-1 IA reference genome.

Experimentally verified genes involved in phytopathogenesis enables the inference of *R. solani* genes for necrotic diseases^15^. Based on sequence similarity with proteins in PHI-base, we identified potential transcripts related to pathogenicity, virulence, and effector genes. Several thousands of contigs in each transcriptome assembly were assigned to any of the relevant categories of phenotype of mutant “Loss of pathogenicity”, “Reduced virulence”, “Increased virulence (Hypervirulence)”, and “Effector (plant avirulence determinant)” (Fig. 2). Among the three AG-1 subgroups, 851 PHI-base accessions were assigned to any of transcriptome contigs in common: these accessions included molecules encode chitin synthase, adenylate cyclase, appressorial penetration, and polyketide synthase (Table S7). This result suggests that the *R. solani* AG-1 genome encodes fundamental pathogenic factors. However, seven PHI-base accessions, for which genes may be associated with greater virulence, were uniquely found in IA-TCs: five bacterial effectors, endochitinase, and pectate lyase (Table S8). These genes may explain the varying pathogenicities and virulence among AG-1 isolates.

Differences in enzyme repertoires among the AG-1 subgroups also reveal insights into the pathogenic mechanism of *R. solani*. We searched KEGG pathways of contigs uniquely found in IA-TCs, IB-TCs, or IC-TCs. While no unique KEGG pathway was found for AG-1 IB and AG-1 IC, AG-1 IA represented four unique pathways including ‘Oxidative phosphorylation’ [KEGG pathway entry: ko00190] (Table S9); this was attributable to mitochondrial proteins such as NADH-quinone oxidoreductase and cytochrome *c* oxidase subunits being uniquely included in IA-TCs. It is reasonable to assume that these genes act on ATP production to support the pathogenicity of AG-1 IA. In addition, we noted that a contig annotated as *1-aminocyclopropane-1-carboxylate synthase* [KEGG pathway entry: K01762], which encodes one of the rate-limiting enzymes in ethylene biosynthesis^43^, was also unique to AG-1 IA. Although the role of pathogen-produced ethylene has been controversial, it is known to promote or suppress disease development in plants^44^. Hoffman *et al*.^45^ reported that ethylene-insensitive soybean mutants exhibit altered susceptibility to a highly virulent *R. solani* strain, depending on their genotype. Pantelides *et al*.^46^ speculated that a necrotrophic pathogen *Fusarium oxysporum* modifies ETR1-mediated ethylene signalling in *Arabidopsis* during disease development.

### Transcripts for phytotoxin biosynthesis

**Figure 3:**
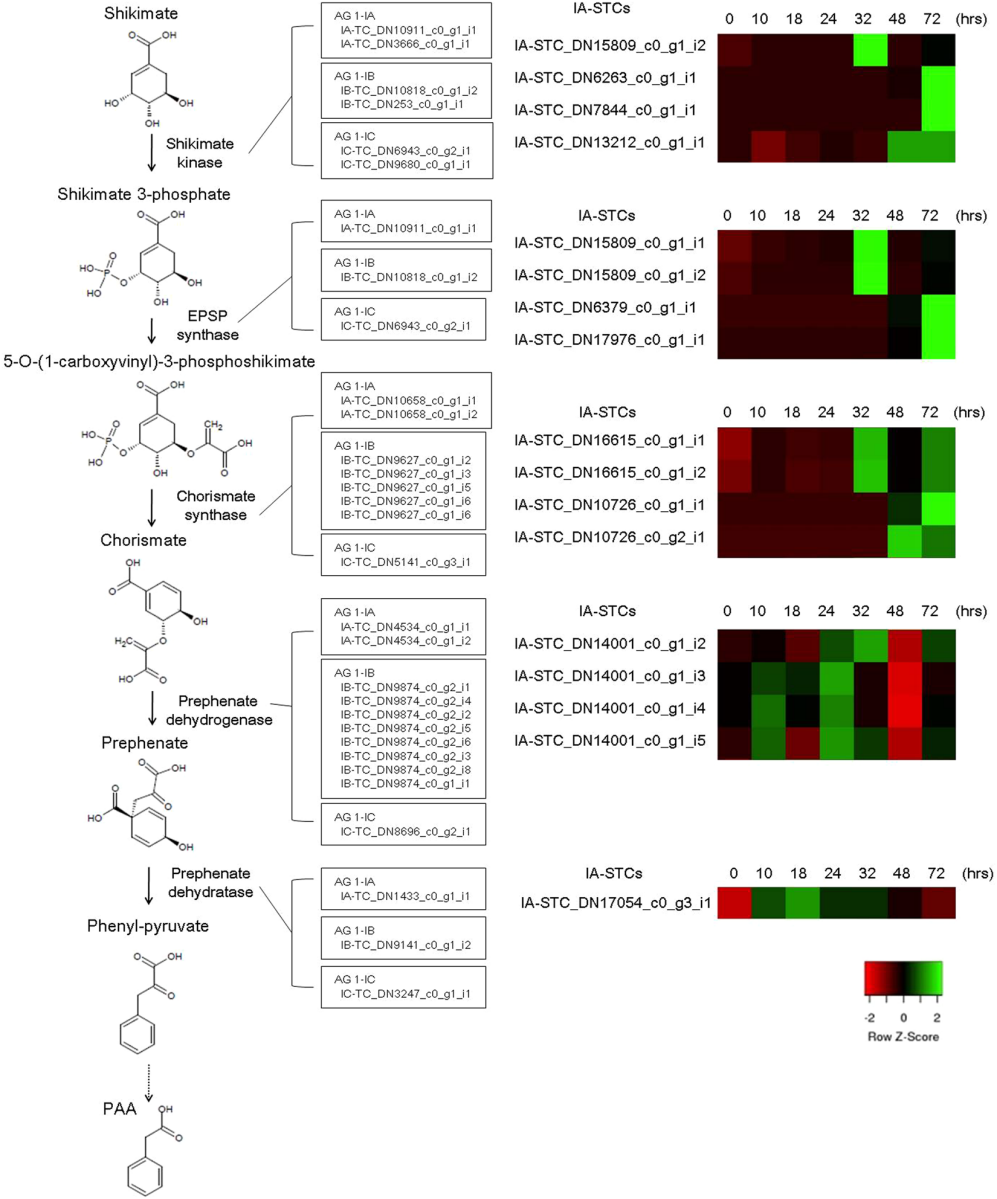
Candidate genes on the biosynthetic pathway for PAA production. Identifiers of transcriptome contigs of IA-TCs, IB-TCs, and IC-TCs were shown in boxes. Expression patterns of candidate genes in IA-STCs (RPKM in log_10_-scale) for this pathway were represented in heatmaps (numerals indicate hours after inoculation onto rice). IA-STC_DN15809_c0_g1_i2 could be annotated as shikimate kinase or EPSP synthase based on the result of the pfam domain search.

PAA is a virulence factor for AG-3 in potato, and the importance of this phytotoxin in sheath blight diseases has been reported in detail^7^. The biosynthetic pathway for PAA has been partially described by Cook *et al*.^47^ based on their experiments in pea. This pathway is initiated by the conversion of shikimate to shikimate 3-phosphate by shikimate kinase [EC 2.7.1.71], followed by four enzymatic reactions catalyzed by 3-phosphoshikimate 1-carboxyvinyltransferase (EPSP synthase) [EC 2.5.1.19], chorismate synthase [EC 4.2.3.5], prephenate dehydrogenase [EC 1.3.1.13], and prephenate dehydratase [EC 4.2.1.51] resulting in the synthesis of phenylpyruvate, which is the probable precursor of PAA. We found that transcript sequences for this pathway commonly existed in IA-TCs, IB-TCs, and IC-TCs (Fig. 3). This is consistent with the notion that AG-1 IA is capable of infecting dicot plant species such as soybean, *Brassica* species, and coffee^5^. Interestingly, we observed obvious induction of three contigs of *shikimate kinase*, two contigs of *3-phosphoshikimate 1-carboxyvinyltransferase*, and one contig of *chorismate synthase* 72 hours after inoculation of AG-1 IA. These transcripts may play roles in the regulation of PAA production.

The metabolite MTPA in AG-3 plays a phytotoxic role during sheath blight because of its effect on membrane integrity in host cells^48^. Possible enzyme-encoding genes for the biosynthetic pathway of MTPA were observed in the transcriptome contigs. Some of the corresponding transcripts appeared to be up-regulated in AG-1 IA after inoculation (Table S10). This observation supported the notion that MTPA is also a pathogenic factor in AG-1 IA.

### Gene sequences for the secretome

The secretion of biologically active proteins is a fundamental strategy employed by fungi when infecting plant host tissues^49^. We predicted secretomes from the transcriptome contigs in two ways: (1) searching protein domains for the classical secretion pathway, and (2) searching protein sequences using EffectorP 2.0^50^, which implements a machine-learning method, and classifying effector candidates into apoplastic or non-apoplastic (cytoplasmic perhaps) using ApoplastP^51^. The total number of potential secretome contigs was 11,581 in IA-TCs, 11,993 in IB-TCs, 9,892 in IC-TCs, and 27,976 in IA-STCs (Table S11). Out of these contigs, 3,046 IA-TCs, 3,060 IB-TCs, 2,509 IC-TCs, and 11,478 IA-STCs were supported by functional annotation, indicating reliability of coding sequence prediction. The predicted secretomes of IA-TCs, IB-TCs, and IC-TCs exhibited similar distributions of KOG classes (Fig. S4); further, we found Pfam protein domains unique to each AG 1 subgroup strain (Table S11). In our analysis, the total number of possible secreted protein genes in AG-1 IA was considerably larger than that (430 gene models) reported by Anderson *et al*.^12^. This would be largely due to the specific secretome prediction methods used. Most of the secretome proteins did not exhibit significant homology with proteins in the fungal secretome database FunSecKB2, implying occurrence of unknown secreted proteins in *R. solani* (Table S11)^52^.

Unexpectedly, several thousands of potential non-apoplastic effector sequences, which may be cytoplasmic effectors, were detected. The conserved motifs for cytoplasmic effectors^50^, RxLR, dEER, and [YFW]xC, were found in the sequences of some of the predicted secreted proteins. The molecular functions of those cytoplasmic effector candidates remained unknown owing to lack of data, although a cytoplasmic effector PsCRN115, which is related to necrosis, was found in *Phytophthora sojae*^53^. There has been no report of cytoplasmic effectors in sheath blight diseases.

The predicted secretomes contained other types of protein homologues that could play roles in promoting colonization of the pathogen. Secreted proteases are thought to contribute to the inactivation of bioactive host proteins released on the surface of tissues and produce low-molecular-weight nitrogen compounds to be absorbed as nutrition. We found sequences for several types of secreted proteases: presenilin aspartyl protease [PF06550.10], eukaryotic aspartyl protease [PF00026.22], papain family cysteine protease [PF00112.22], deuterolysin metalloprotease [PF02102.14], CAAX prenyl protease [PF16491.4], and ATP-dependent protease [PF02190.15]. In total, 73 IA-STCs coding protease homologues were found, and 61 of them were expressed in the mycelia before inoculation onto rice. The remaining 12 contigs were inducible at 32 hours after inoculation. These two expression patterns may indicate different physiological roles. CAZymes in *R. solani* have often been described in genome and transcriptome studies^4,10,54^ because the degradation of plant cell wall components can be critical for expansion of mycelia and production of nutrients. Various CAZymes were included in the secretomes for the AG-1 subgroups (Table S11). With regards to IA-STCs, 521 contigs were annotated as secreted CAZymes across all the major CAZymes classes^40^: auxiliary activities (AAs), carbohydrate esterases (CEs), glycoside hydrolases (GHs), glycosyltransferases (GTs), and polysaccharide lyases (PLs).

We also found gene sequences annotated as reactive oxygen species (ROS) detoxification enzymes: *catalase*, *glutaredoxin*, *glutathione peroxidase*, *glutathione S-transferase*, *copper/zinc superoxide dismutase*, and *iron/manganese superoxide dismutase* in the secretomes. It is possible that these enzymes ROS produced in accordance with PCD. These proteins were frequently observed in the secretomes of the mycelial transcriptome contigs (Table S12). Some enzyme genes for the glutathione system exhibited higher expression levels after inoculation of AG-1 IA than before inoculation (data not shown). Ghosh *et al*.^54^ described the potential involvement of an NifU-like protein in *R. solani* cells in detoxifying ROS produced by host plants. In addition, an RNA-Seq study^55^ reported that differential transcriptional regulation during ROS production was observed in two rice varieties showing contrasting sheath blight resistance; in fact, the expression levels of an *NADPH oxidase* and two *oxalate oxidases*, which are key respiratory burst oxidases for ROS production^56^, were concordant with colonization by this fungus. These results support the concept that secreted ROS detoxification enzymes play an indirect role in development of necrotic lesions.

### Small secreted proteins

**Figure 4:**
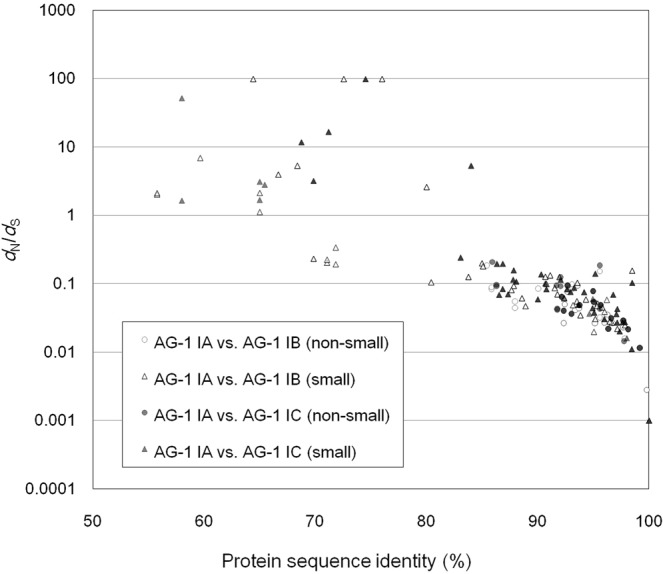
Relationship of protein sequence similarity and *d*_N_/*d*_S_ ratios of 123 putative orthologous secreted proteins among AG-1. This protein set was based on the search by cd-hit-2d, a subtool of CD-HIT^64^. The horizontal axis and the vertical axis represent protein sequence identify and *d*_N_/*d*_S_, respectively. White and gray circles indicate pair-wise comparisons of non-small (no short than 300 amino acids) secreted proteins between AG-1 IA and AG-1 IB and AG-1 IA and AG-1 IC, respectively. White and gray triangles indicate pair-wise comparisons of small secreted proteins between AG-1 IA and AG-1 IB and AG-1 IA and AG-1 IC, respectively.

Transcriptome-wide secretome comparisons among the AG-1 subgroups could provide insights into the molecular evolution of *R. solani*. To conduct a pilot examination, we clustered the predicted secreted protein sequences into probable orthologous groups and found 123 trios of secreted proteins, which enabled the inference of one-to-one relationships between IA-TCs, IB-TCs, and IC-TCs under a conservative criterion [no less than 50% of amino acid sequence identity and within 20% of protein length difference]. Mapping of the secretome sequences on genome assemblies for AG-1 subgroups supported most of (95%~) orthologous relationships in the 123 trios, indicating the appropriateness of this criterion for evolutionary analyses. The majority of the 123 secreted protein trios showed high sequence conservation with over 85% amino acid sequence identity as well as small *d*_N_ (the number of non-synonymous substitutions per site)/*d*_S_ (the number of synonymous substitutions per site) ratios of less than 1 (Fig. 4), implying that these protein genes were under purifying selection pressure after speciation of the AG-1 subgroups and essential components in this pathogen. In fact, these slow-evolving proteins included homologues of a protein transport protein SEC61 subunit alpha, a chitin synthase export chaperone CHS7, a protein mannosyltransferase and two effector candidates. In contrast, fast-evolving proteins, which exhibited less than 85% amino acid sequence identity, represented *d*_N_/*d*_S_ ratios of more than 1 in most cases. These proteins may have been under positive or relaxed selection pressure after divergence of the AG-1 subgroups. Importantly, these fast-evolving proteins with high *d*_N_/*d*_S_ ratios were ‘small secreted proteins’ comprised of no longer than 300 amino acids. Here, we noted that cysteine-rich small secreted proteins (CSSPs, no less than 4% of cysteine) were enriched in the fast-evolving protein category (35.3%) compared with that in all 123 trios (13.8%) with statistical significance (*p* value of 0.079 in Fisher’s exact test), while small secreted proteins were not obviously enriched. This result indicates the significance of CSSPs in molecular diversification of the secretomes in AG-1. CSSPs can directly interact with environmental factors and molecules of host plants or microbiomes; therefore, fast-evolving CSSPs under positive selection pressure are likely to play specific roles in the life cycle of *R. solani*. Fast-evolving CSSPs under relaxed selection pressure may have lost critical roles during their recent evolution; therefore, such CSSP genes may represent a genetic reservoir for new molecular function. It is noteworthy that none of 21 effector candidate trios exhibited *d*_N_/*d*_S_ ratios of over 1.

CSSPs have been at the center of studies of molecular interactions between phytopathogens and host plants; the physical properties resulting from protein sizes and Cys-richness substantiate higher probabilities of biological significance^57^. We identified 1,872 IA-TCs, 2,009 IB-TCs, 1,555 IC-TCs, and 7,446 IA-STCs that encode CSSPs (Table S10). These contained homologues of expansin, pathogenesis-related protein 5, and cellulase, which are likely to be involved in pathogenicity^58–60^. Proteins homologous to γ-interferon-inducible lysosomal thiol reductase and CFEM domain-containing protein were uniquely found in AG-1 IA. However, no functional annotation was given to most of the CSSPs contigs, implying that these represent candidate pathogenic factors. To assess the possibility that these non-annotated protein genes are effectors, we conducted a bioassay by injecting *E. coli* recombinant proteins encoded by CSSP genes into detached rice leaves (Table S13). An orthologous gene pair induced obvious necrosis (Fig. S5). The primary structure of this orthologous protein and Cys residues was highly conserved among AG-1 (Fig. S6). We noted that these effector candidates contained glycine and serine residues at high rates (13.7–14.7 and 9.3–12.0%, respectively). These small amino acids and Cys are likely to contribute to the compactness of the proteins, which confers high mobility in the apoplast. Prediction of allergenicity by AllerCatPro^61^ suggested that these candidate effector proteins are homologous to an allergenic protein Mala g 8 of the skin pathogen *Malassezia globosa*. Interestingly, this protein in AG-1 IA did not elicit necrosis in rape and soybean leaves (data not shown), and agroinfiltration of this gene on tobacco leaves did not induce necrosis (unpublished result), implying a host-range limitation of this effector candidate.

### Differentially regulated transcriptome contigs in response to inoculation onto rice

**Figure 5:**
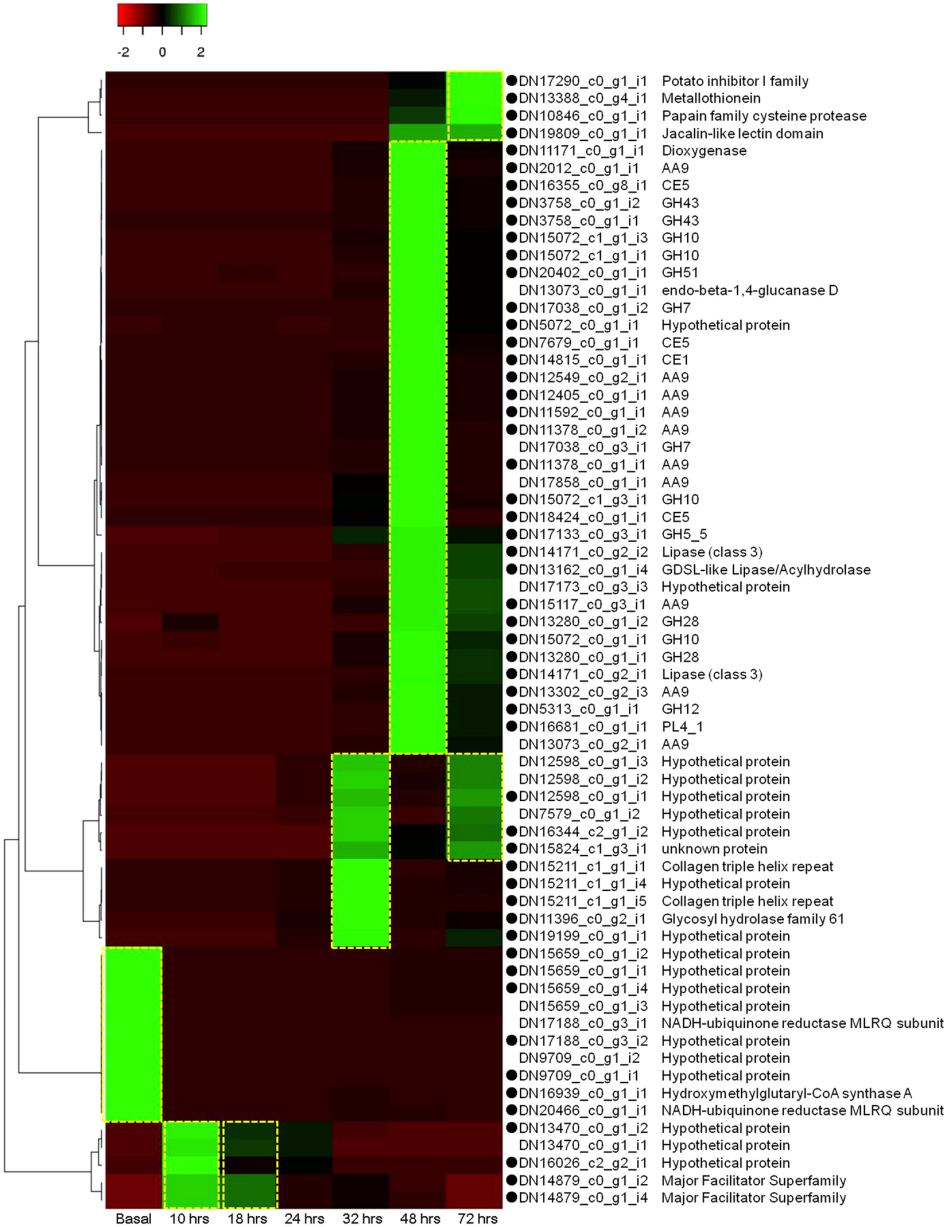
Graphical representation of differentially regulated genes by inoculation of the AG-1 IA strain. The heat map represents expression patterns of genes in log_10_-scale. The expression patterns were grouped into five clusters using the complete linkage of Pearson correlation coefficient. Black boxes in front of the contig identifiers indicate probable secreted protein gene transcripts. Short functional annotations of the contigs were shown in the right of the contig identifiers. Dot boxes in yellow indicate up-regulated clusters.

Gene expression patterns can be powerful clues when identifying genes related to biological phenomena. In total, 65 differentially regulated contigs were identified with statistical significance (10% level) by pair-wise comparison between the basal profile and mycelial profiles after inoculation. Those contigs included 55 up-regulated contigs after inoculation and 10 down-regulated ones. The up-regulated contigs included CAZymes, lipases, a protease, a dioxygenase, a jacalin-like lectin, and other proteins (Fig. 5). CAZymes were up-regulated in the sample of 48 hours after inoculation, at which post-infection processes occur. Our results indicate the importance of secreted CAZymes and highlight the roles of CE1 and CE5, which preferentially catalyze the deacetylation of 2-*O* acetyl 4-nitrophenyl β-D-xylopyranoside (Fig. 5). Ten contigs exhibited up-regulated expression patterns within 24 hours after inoculation (Fig. 5). For example, contigs of the major facilitator family protein exhibited enhanced expression at 10 and 18 hours after inoculation, but down-regulation at 24 hours after inoculation (Fig. S7). Interestingly, this major facilitator transporter was annotated as a secreted protein. A major facilitator transporter in a pathogenic fungus *Cercospora* plays a role in the biosynthesis of the non-selective phytotoxin cercosporin^62^. The down-regulated contigs contained proteins annotated as hydroxymethylglutaryl-CoA synthase A and NADH-ubiquinone reductase complex 1 MLRQ subunit. The constant expression of these genes after inoculation suggests that they might be involved in fundamental biological processes.

**Figure 6:**
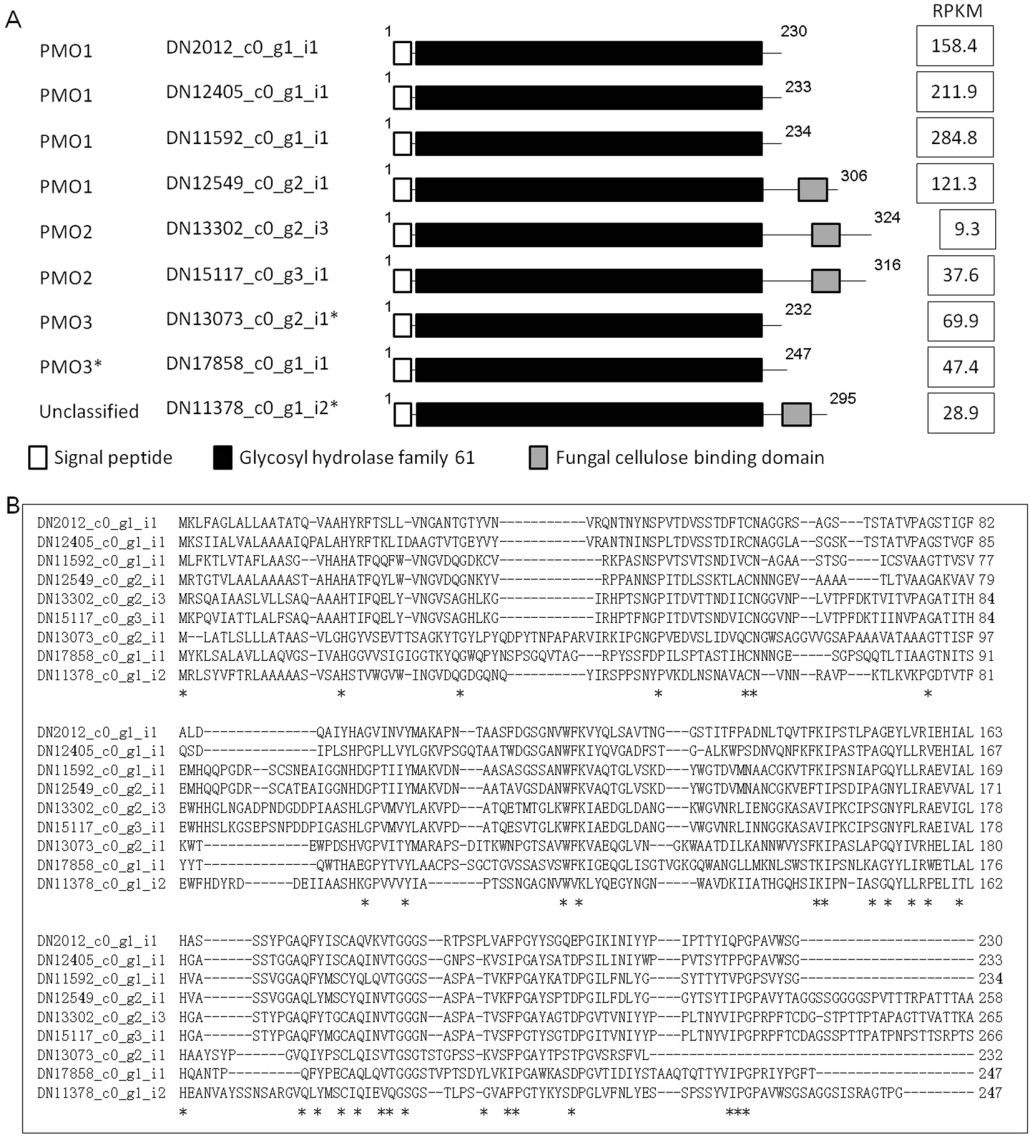
Structure and gene expression levels of AA9 in the up-regulated cluster of 48 hours after inoculation. (**A**) graphical representation of AA9 proteins and RPKM. Asterisks with the contig identifier indicate that part of coding sequences were recovered from the AG-1 IA draft genome sequence. (**B**) Partial sequence alignment of AA9 proteins. The N-terminus and protein domain for glycosyl hydrolase family 61 were shown. Asterisks indicate completely identical amino acid residues among the AA9 proteins.

Gene Ontology (GO) enrichment analysis of the seven up-regulated contig clusters revealed biological terms related to lignocellulose degradation at 48 hours after inoculation (Table S14). This was attributable to enrichment of CAZyme contigs with statistical significance (*p* value = 0). AA9, which plays a central role in the oxidative degradation of cellulose^63^, was present in the highest number among the CAZymes in the cluster. These AA9 contigs corresponded to nine genomic genes that encode secreted AA9 proteins (Fig. 6). The AA9 proteins were categorized into the four major groups, PMO1, PMO2, PMO3, and PMO3*, and exhibited structural diversification including presence/absence of the carbohydrate-binding domain. Gene expression levels of *AA9* were also divergent, and *AA9* in the PMO1 group exhibited highest expression levels than in other groups. Homologous proteins of these AA9 proteins were found in IB-TCs, IC-TCs, as well as in AG-3 and AG-8. Molecular diversity of AA9 may be essential in pathogenesis of *R. solani*. We mined six motifs in the 1-kb upstream region of the coding sequences of the AA9 (Fig. S8). Motif 1 to motif 4 were not found for any of other 11 secreted AA9 contigs, implying that these four motifs were involved in induction of *AA9*. The absence of these motifs in other secreted *CAZymes* in the same cluster suggests that the *AA9* genes are regulated independently from other *CAZymes*. To our knowledge, other than the present findings, cis-elements for fungal *CAZymes* have not been reported to date.

## Methods

### Fungal materials

Three *R. solani* strains obtained from South China Agricultural University, the national standard isolate of AG-1 IA^10^, and one isolate each for AG-1 IB, and AG-1 IC, were used. Microscopic observations indicated that these strains had multinucleate cells (data not shown). Sclerotia were germinated and mycelia were inoculated on potato dextrose broth in bottles and cultured at 28 °C under dark conditions with shaking at 150 rpm for two days. Mycelia from culture replicates were harvested and quickly dried after washing with sterile deionized water.

### RNA-Seq

Total RNAs were extracted from mycelia using the E.Z.N.A. Fungal RNA Kit (Omega Bio-teck, Inc., GA, USA), according to the manufacturer’s instructions. Then, the RNA fractions were incubated with RNase-free DNase I at 37 °C for 30 min to remove genomic DNA contaminants. Purity and quantity of the total RNAs were determined using NanoDrop ND-1000 (Thermo Scientific, Inc., MA, USA). RNA integrity was verified by agarose electrophoresis.

mRNA sequencing was carried out by the BGI Group (Shenzhen, China). Poly (A)^+^ RNAs were selected using poly-T attached magnetic beads and then randomly fragmented in 1 × fragmentation buffer (Affymetrix, Inc., CA, USA). The fragmented poly (A)^+^ RNA fractions were used for construction of cDNA libraries. Briefly, random hexamer primers were annealed with the fragmented RNAs, and first-strand cDNAs were synthesized and converted into double-stranded cDNAs. Then, the termini of the cDNAs were end-repaired and ligated with adaptors after adenylation at the 3′-ends. After gel purification, the cDNAs were amplified by PCR and analyzed by an Illumina GA II sequencer (Illumina, CA, USA) to generate 72 bp × 75 bp paired-end sequences. The raw sequence data were deposited in the NCBI Sequencing Read Archive (SUB5156870).

### Transcriptome assembly

Raw sequence reads of each strain were independently assembled into transcriptome contigs. Trinity^17^ version 2.6.5 and Velvet version 1.2.10/Oases^18^ version 0.2.09 were used for optimization of assembly. The options ‘–trimmomatic’ and ‘–jaccard_clip’ for *de novo* assembly and genome-guided assembly in Trinity with the default parameters were tested. For Velvet/Oases, different *k*-mer from 13 to 71 with the default parameters were applied. The best assembly condition, of Trinity with the option ‘–trimmomatic’, was selected based on evaluation by BUSCO^64^ version 3.0.2 with the option of ‘-m tran’, in which the two lineage data sets for fungi and Basidiomycota were applied to verify the existence of common genes in the assembled sequences. The raw sequence reads of AG-1 IA and previously generated mRNA sequencing data of AG-1 IA after inoculation onto rice leaves^10^ were also assembled into super-transcriptome contigs using Trinity, with the abovementioned best condition. Relationships among the transcriptome contigs in AG-1 IA were designated by CD-HIT^65^ version 4.6.8 with the option of ‘-c 0.95 -n 10’.

### Sequence comparisons

Sequence similarity between genomic DNA and transcript sequences was analyzed by BLAT^19^ version 36. Similarity between transcript sequences was analyzed by BLASTN of BLAST^+^ version 2.4.0 with the E-value of 1e-5^20^. Similarity between predicted protein sequences was analyzed by BLASTP of BLAST^+^. Protein sequences of *R. solani* AG-3, AG-8, *Magnaporthe oryzae*, *F. oxysporum*, *Botrytis cinerea*, *Colletotrichum higginsianum*, *Moniliophthora perniciosa*, *Phaeosphaeria nodorum*, *Sclerotinia sclerotiorum*, and *Ustilago maydis* were downloaded from Ensembl Fungi (http://fungi.ensembl.org/index.html) version 2018.6.22, and those of *L. bicolor* and *C. cinerea* were downloaded from the JGI Genome Portal^66^.

Divergence time was estimated using the protein sequences of α-tubulin, cell division control protein 42, ras-related nuclear protein, eukaryotic translation initiation factor, heat shock 70 kDa protein, and β-tubulin. A multiple sequence alignment for the concatenated sequences were prepared by MUSCLE, and a phylogenetic tree was constructed by the method of maximum likelihood with the Dayhoff, JTT, LB, and WAG model implemented in MrBayes version 3.2^67^. *Phanerochaete* chrysosporium, *C. cinerea*, *Puccinia graminis* f. *U. maydis*, *Cryptococcus neoformans*, and *M. oryzae* were used as references. *Saccharomyces cerevisiae* was used as the outgroup.

### Mining of SSRs on transcripts

SSRs on the mycelial transcriptome assemblies were detected using Phobos version 3.3.12 (http://www.rub.de/ecoevo/cm/cm_phobos.htm) with the options ‘–searchMode extendExact -u 2 -U 1000’.

### Mining of multinucleate polymorphisms

Intra-strain polymorphic sites were mined as follows; (1) RNA-Seq reads were re-mapped on the mycelial transcriptome contigs using BWA mem^68^ version 0.7.15 with the default condition, (2) re-mapping data were converted into bam files and sorted using the SAMtools^69^ version 1.9, (3) local read alignments were corrected by Genome Analysis Toolkit^70^ version 3.7, (4) realigned data were converted into mpileup format by SAMtools with the option ‘-q 13’, (5) nucleotide substitutions were detected by SNiPer3, which is a derivative tool of a variant caller SNiPer2^22^. Alternative nucleotide frequencies of no less than 5%, with a minimum of two reads supporting each allele, were detected, (6) short insertions and deletions were called by VarScan^71^ version 2.4.0 with the default conditions, and (7) to filter out false positive sites caused by technical errors in the transcriptome sequencing processes, the threshold of the minimum read depth of 20 and allelic concordance of 20%, which seemed reliable based on a small-scale comparison to genome-based polymorphic sites of 100 sites for AG-1 IA (Fig. S9), were applied. For adjusting different sequencing depth among the AG-1 subgroups, the minimum read depth of 19 for AG-1 IB and 16 for AG-1 IC were applied. Sequential nucleotide substitution sites were merged into potential multi-nucleotide polymorphisms. For referential data, genome-based multinucleate polymorphisms in AG-1 IA were mined using the genomic short reads and the reference genomic scaffolds described elsewhere^10^ with the same re-mapping strategy as above.

### Gene annotation

Coding sequences of the transcriptome contigs were predicted by TransDecoder^17^ version 5.3.0 with an option ‘-m 50’. All predicted coding sequences were analyzed by HMMER^72^ version 3.2.1 to predict protein domains of the Pfam database^34^ version 31.0. Metabolic pathway annotations were given by the KEGG Automatic Annotation Server with the SBH method^73^. Annotation of CAZymes were given by dbCAN^74^ with HMMdb v7.0 under the default setting parameters (E-value < 1e-15 and coverage >0.35). KOGs^35^ were assigned by BLASTP searches with the E-value of 1e-5. Homologous protein sequences were searched against protein sequences of the PHI-base version 4.6^38^ and the protein database Swiss-Prot^36^ by BLASTP searches with an E-value of 1e-5.

Secreted proteins were inferred from predicted coding sequences based on presence or absence of probable signal peptides, transmembrane domains, *ω*-sites for glycosylphosphatidylinositol (GPI) anchor, and transit peptides to mitochondrion. SignalP 4.1^75^ with the D-cutoff value of 0.34, PrediSi^76^ and Phobius^77^ were used to predict signal peptides, PrediSi, Phobius, and TMHMM^75^ version 2.0c were used to predict transmembrane domains, and proteins with mitochondrial transit peptides were predicted using TargetP^75^ version 1.1. PredGPI was used to annotate *ω*-sites^78^. A secreted protein based on signal peptide prediction, absence of transmembrane domain in the N-terminal of the signal peptide, the protein not being targeted to the mitochondrion, and absence of *ω*-sites. *d*_N_/*d*_S_ ratios of probable full-length orthologous proteins were measured using PAL2NAL^79^ with the default parameters.

Effector candidates were searched from among the predicted coding sequences of the transcriptome contigs using EffectorP 2.0^50^. Localization of probable effectors was predicted using the ApoplastP^51^ version 1.0.1. Sequences of predicted secretome proteins were searched against the fungal secretome database FunSecKB2^52^ using BLASTP with the E-value of 1e-2 for annotation.

### Quantification of gene expression levels

RNA-Seq reads were re-mapped on the super-transcriptome contigs using Bowtie2^80^ version 2.3.4 with the default condition, and mapping data (bam files) were generated to count reads on each contig using SAMtools. Read counts of contigs were normalized to identify differentially expressed genes with the false discovery rate of no more than 0.1 by TCC^81^ with three-time iterations by DESeq2 (denoted as ‘iDEGES/DESeq2-DESeq2’)^82^.

### Expression of recombinant proteins

cDNAs of effector candidate were amplified from 1st-stranded cDNA fractions of the AG-1 strains by RT-PCR using gene-specific primers (Table S14). The PCR products were cloned into pMD19-T simple (Takara Bio Inc., Japan), and then the cDNA inserts were subcloned into an expression vector, pCzn1 (Zoonbio Biotechnology Co. Ltd., China) or qQE-30 (Qiagen, Germany). The expression constructs of pCzn1 were transformed into an *E. coli* strain ArcticExpress^TM^ (DE3), then recombinant proteins were expressed by induction of isopropyl β-D-1-thiogalactopyranoside (IPTG). Expressed proteins were extracted with 10-fold weight of 0.1 mM phenylmethylsulfonyl fluoride by boiling for a few minutes. Cell debris was removed by centrifugation. The expression constructs of qQE-30 were transformed into the *E. coli* strain JM109, and then recombinant proteins were induced by IPTG. Soluble proteins were extracted by the same method with pCzn1. Supernatants were analyzed in SDS-PAGE by the method of Laemmli^83^.

### Bioassays for effector candidates

Rice plants of variety Teqing were grown in a growth chamber for a few weeks. Second leaves of the plants were used for infiltration of recombinant proteins. For the mock experiments, leaves were treated with sterile deionized water. Recombinant proteins (several μg of extracted protein from transformed *E. coli* cell) were infiltrated into detached leaves and incubated at 28 °C under 80% humidity. After three days, necrotic states were monitored visually.

### GO enrichment analysis

GO terms were given to transcriptome contigs based on Pfam domain annotation. GO terms in each up-regulated IASTC cluster and in all the IASTCs were counted, and enrichment of GO terms were statistically analyzed tested by Fisher’s exact test with the Bonferroni correction.

### Cis-motif search

Upstream regions (1 kb) of coding sequences were retrieved from the AG-1 IA reference genome sequence. Common short sequence motifs were searched using MEME suit version 5.0.5^84^.

## Supplementary information

Table S1

Table S2

Table S3

Table S4

Table S5

Table S6

Table S7

Table S8

Table S9

Table S10

Table S11

Table S12

Table S13

Table S14

Table S15

Supplementary Figures

## Data Availability

The Perl script SNiPer3, transcriptome contig sequences, predicted protein sequences, and the functional annotations are available upon request.

## References

[1] Ogoshi A (1987). Ecology and Pathogenicity of Anastomosis and Intraspecific Groups of *Rhizoctonia Solani* Kuhn. Ann. Rev. Phytopathol..

[2] Vidhyasekaran P (1997). Host-Specific Toxin Production by *Rhizoctonia solani*, the Rice Sheath Blight Pathogen. Phytopathology.

[3] Yang GH, Conner RL, Chen YY, Chen JY, Wang YG (2008). Frequency and pathogenicity distribution of *Rhizoctona* spp. causing sheath blight on rice and banded leaf disease on Maize in Yunnan, China. J. Plant Pathol..

[4] Taheri P, Tarighi S (2011). Cytomolecular aspects of rice sheath blight caused by *Rhizoctonia solani*. Eur. J. Plant Pathol..

[5] Jones RK, Belmar SB (1989). Characterization and pathogenicity of *Rhizoctonia* spp. isolated from rice, soybean, and other crops grown in rotation with rice in Texas. Plant Disease.

[6] Chauhan, S. B., Jabran, K. & Mahajan, G. Rice Production Worldwide, Springer International USAISBN: 978-3-319-47516-5 (2017).

[7] Brooks SA (2007). Sensitivity to a Phytotoxin from *Rhizoctonia solani* Correlates with Sheath Blight Susceptibility in Rice. Phytopathology.

[8] Hu W, Pan X, Li F, Dong W (2018). UPLC-QTOF-MS metabolomics analysis revealed the contributions of metabolites to the pathogenesis of *Rhizoctonia solani* strain AG-1-IA. PLoS One.

[9] Lakshman DK, Alkharouf N, Roberts DP, Natarajan SS, Mitra A (2012). Gene expression profiling of the plant pathogenic basidiomycetous fungus *Rhizoctonia solani* AG 4 reveals putative virulence factors. Mycologia.

[10] Zheng A (2013). The evolution and pathogenic mechanisms of the rice sheath blight pathogen. Nat. Commun..

[11] Wibberg D (2014). Transcriptome analysis of the phytopathogenic fungus *Rhizoctonia solani* AG1-IB 7/3/14 applying high-throughput sequencing of expressed sequence tags (ESTs). Fungal Biol..

[12] Anderson JP (2017). Comparative secretome analysis of *Rhizoctonia solani* isolates with different host ranges reveals unique secretomes and cell death inducing effectors. Sci. Rep..

[13] Dickman MB, de Figueiredo P (2013). Death be not proud-cell death control in plant fungal interactions. PLoS Pathog..

[14] Shibu MA, Lin HS, Yang HH, Peng KC (2012). Trichoderma harzianum ETS 323-mediated resistance in *Brassica oleracea* var. capitata to *Rhizoctonia solan*i involves the novel expression of a glutathione S-transferase and a deoxycytidine deaminase. J. Agric. Food Chem..

[15] Wibberg D (2015). Development of a *Rhizoctonia solani* AG1-IB Specific Gene Model Enables Comparative Genome Analyses between Phytopathogenic *R. solani* AG1-IA, AG1-IB, AG3 and AG8 Isolates. PLoS One.

[16] Wibberg D (2016). Genome analysis of the sugar beet pathogen *Rhizoctonia solani* AG2-2IIIB revealed high numbers in secreted proteins and cell wall degrading enzymes. BMC Genomics.

[17] Haas BJ (2013). *De novo* transcript sequence reconstruction from RNA-seq using the Trinity platform for reference generation and analysis. Nat. Protoc..

[18] Schulz MH, Zerbino DR, Vingron M, Birney E (2012). Oases: robust *de novo* RNA-seq assembly across the dynamic range of expression levels. Bioinformatics.

[19] Kent WJ (2002). BLAT-the BLAST-like alignment tool. Genome Res..

[20] Altschul SF, Gish W, Miller W, Myers EW, Lipman DJ (1990). Basic local alignment search tool. J. Mol. Biol..

[21] Rokas A, Krüger D, Carroll SB (2005). Animal evolution and the molecular signature of radiations compressed in time. Science.

[22] Kuninaga S, Yokosawa R (1982). DNA Base Sequence Homology in *Rhizoctonia solani* Kuhn I. Genetic relatedness within anastomosis group 1. Ann. Phytopath. Soc. Japan.

[23] Vilgalys R (1988). Genetic relatedness among anastomosis groups in *Rhizoctonia* as measured by DNA/DNA hybridization. Phytopathology.

[24] Yamamoto N (2018). Comparative whole genome re-sequencing analysis in upland New Rice for Africa: insights into the breeding history and respective genome compositions. Rice.

[25] Gilbert, D. G. Genes of the Pig, Sus scrofa, reconstructed with EvidentialGene. *BioRxiv*, 412130 (2018) 10.7717/peerj.6374 PMC6361002 30723633

[26] Li YC, Korol AB, Fahima T, Nevo E (2004). Microsatellites within genes: structure, function, and evolution. Mol. Biol. Evol..

[27] Varshney RK (2005). Interspecific transferability and comparative mapping of barley EST-SSR markers in wheat, rye and rice. Plant Sci..

[28] Endo C (2017). Development of simple sequence repeat markers in the halophytic turf grass *Sporobolus virginicus* and transferable genotyping across multiple grass genera/species/genotypes. Euphytica.

[29] Tao SQ, Cao B, Tian CM, Liang YM (2018). Development and Characterization of Novel Genic-SSR Markers in Apple-Juniper Rust Pathogen *Gymnosporangium yamada*e (Pucciniales: Pucciniaceae) Using Next-Generation Sequencing. Int. J. Mol. Sci..

[30] Roper M, Ellison C, Taylor JW, Glass NL (2011). Nuclear and genome dynamics in multinucleate ascomycete fungi. Curr. Biol..

[31] Grosch R, Schneider JHM, Kofoet A (2004). Characterisation of *Rhizoctonia solani* anastomosis groups causing bottom rot in field-grown lettuce in Germany. Eur. J. Plant Pathol..

[32] Hane JK, Anderson JP, Williams AH, Sperschneider J, Singh KB (2014). Genome sequencing and comparative genomics of the broad host-range pathogen *Rhizoctonia solan*i AG8. PLoS Genet..

[33] Au CH (2013). Rapid genotyping by low-coverage resequencing to construct genetic linkage maps of fungi: a case study in *Lentinula edodes*. BMC Res. Notes.

[34] Hacquard S (2013). Mosaic genome structure of the barley powdery mildew pathogen and conservation of transcriptional programs in divergent hosts. Proc. Natl. Acad. Sci. USA.

[35] Tatusov RL (2003). The COG database: an updated version includes eukaryotes. BMC Bioinformatics.

[36] Boutet E (2016). UniProtKB/Swiss-Prot, the Manually Annotated Section of the UniProt KnowledgeBase: How to Use the Entry View. Methods Mol. Biol..

[37] Finn RD (2014). Pfam: the protein families database. Nucleic Acids Res..

[38] Kanehisa M, Sato Y, Kawashima M, Furumichi M, Tanabe M (2016). KEGG as a reference resource for gene and protein annotation. Nucleic Acids Res..

[39] Urban M (2017). PHI-base: a new interface and further additions for the multi-species pathogen-host interactions database. Nucleic Acids Res..

[40] Lombard V, Golaconda Ramulu H, Drula E, Coutinho PM, Henrissat B (2013). The carbohydrate-active enzymes database (CAZy) in 2013. Nucleic Acids Res..

[41] Kochetov AV (2008). Alternative translation start sites and hidden coding potential of eukaryotic mRNAs. Bioessays.

[42] Mouilleron H, Delcourt V, Roucou X (2016). Death of a dogma: eukaryotic mRNAs can code for more than one protein. Nucleic Acids Res..

[43] Lin Z, Zhong S, Grierson D (2009). Recent advances in ethylene research. J. Exp. Bot..

[44] van Loon LC, Geraats BP, Linthorst HJ (2006). Ethylene as a modulator of disease resistance in plants. Trends Plant Sci..

[45] Hoffman T, Schmidt JS, Zheng X, Bent AF (1999). Isolation of ethylene-insensitive soybean mutants that are altered in pathogen susceptibility and gene-for-gene disease resistance. Plant Physiol..

[46] Pantelides IS, Tjamos SE, Pappa S, Kargakis M, Paplomatas EJ (2013). The ethylene receptor ETR1 is required for *Fusarium oxysporum* pathogenicity. Plant Pathol..

[47] Cook SD (2016). Auxin Biosynthesis: Are the Indole-3-Acetic Acid and Phenylacetic Acid Biosynthesis Pathways Mirror Images?. Plant Physiol..

[48] Kankam F (2016). 3-Methylthiopropionic Acid of *Rhizoctonia solan*i AG-3 and Its Role in the Pathogenicity of the Fungus. Plant Pathol. J..

[49] Lo Presti L (2015). Fungal effectors and plant susceptibility. Annu. Rev. Plant Biol..

[50] Sperschneider J, Dodds PN, Gardiner DM, Singh KB, Taylor JM (2018). Improved prediction of fungal effector proteins from secretomes with EffectorP 2.0. Mol. Plant. Pathol..

[51] Sperschneider J, Dodds PN, Singh KB, Taylor JM (2018). ApoplastP: prediction of effectors and plant proteins in the apoplast using machine learning. New Phytol..

[52] Lum G, Min XJ (2011). FunSecKB: the Fungal Secretome KnowledgeBase. Database.

[53] Zhang M (2015). A *Phytophthora sojae* cytoplasmic effector mediates disease resistance and abiotic stress tolerance in *Nicotiana benthamiana*. Sci. Rep..

[54] Ghosh S, Gupta SK, Jha G (2014). Identification and functional analysis of AG1-IA specific genes of *Rhizoctonia solani*. Curr. Genet..

[55] Zhang J (2017). Comparative Transcriptome Analyses of Gene Expression Changes Triggered by *Rhizoctonia solani* AG1 IA Infection in Resistant and Susceptible Rice Varieties. Front. Plant Sci..

[56] Marino D (2012). A burst of plant NADPH oxidases. Trends Plant Sci..

[57] Stergiopoulos I, de Wit PJ (2009). Fungal effector proteins. Annu. Rev. Phytopathol..

[58] van Loon LC, van Strien EA (1999). The families of pathogenesis-related proteins, their activities, and comparative analysis of PR-1 type proteins. Physiol. Mol. Plant P..

[59] Liu J (2016). Molecular Characterization of A Novel Effector Expansin-like Protein from *Heterodera avenae* that Induces Cell Death in *Nicotiana benthamiana*. Sci. Rep..

[60] Ma Z (2017). A paralogous decoy protects *Phytophthora sojae* apoplastic effector PsXEG1 from a host inhibitor. Science.

[61] Maurer-Stroh S (2019). AllerCatPro―Prediction of protein allergenicity potential from the protein sequence. Bioinformatics.

[62] de Jonge R (2018). Gene cluster conservation provides insight into cercosporin biosynthesis and extends production to the genus. Colletotrichum. Proc. Natl. Acad. Sci. USA.

[63] Vu VV, Beeson WT, Phillips CM, Cate JH, Marletta MA (2014). Determinants of regioselective hydroxylation in the fungal polysaccharide monooxygenases. J. Am. Chem. Soc..

[64] Simão FA, Waterhouse RM, Ioannidis P, Kriventseva EV, Zdobnov EM (2015). BUSCO: assessing genome assembly and annotation completeness with single-copy orthologs. Bioinformatics.

[65] Fu L, Niu B, Zhu Z, Wu S, Li W (2012). CD-HIT: accelerated for clustering the next-generation sequencing data. Bioinformatics.

[66] Grigoriev IV (2012). The genome portal of the Department of Energy Joint Genome Institute. Nucleic Acids Res..

[67] Ronquist F (2012). MrBayes 3.2: efficient Bayesian phylogenetic inference and model choice across a large model space. Syst. Biol..

[68] Li H, Durbin R (2009). Fast and accurate short read alignment with Burrows-Wheeler transform. Bioinformatics.

[69] Li H (2009). 1000 Genome Project Data Processing Subgroup. The Sequence Alignment/Map format and SAMtools. Bioinformatics.

[70] McKenna A (2010). The Genome Analysis Toolkit: a MapReduce framework for analyzing next-generation DNA sequencing data. Genome Res..

[71] Koboldt DC (2012). VarScan 2: somatic mutation and copy number alteration discovery in cancer by exome sequencing. Genome Res..

[72] Eddy SR (1998). Profile hidden Markov models. Bioinformatics.

[73] Moriya Y, Itoh M, Okuda S, Yoshizawa AC, Kanehisa M (2007). KAAS: an automatic genome annotation and pathway reconstruction server. Nucleic Acids Res..

[74] Zhang H (2018). dbCAN2: a meta server for automated carbohydrate-active enzyme annotation. Nucleic Acids Res..

[75] Emanuelsson O, Brunak S, von Heijne G, Nielsen H (2007). Locating proteins in the cell using TargetP, SignalP and related tools. Nat Protoc..

[76] Hiller K, Grote A, Scheer M, Münch R, Jahn D (2004). PrediSi: prediction of signal peptides and their cleavage positions. Nucleic Acids Res..

[77] Käll L, Krogh A, Sonnhammer EL (2004). A combined transmembrane topology and signal peptide prediction method. J. Mol. Biol..

[78] Pierleoni A, Martelli PL, Casadio R (2008). PredGPI: a GPI-anchor predictor. BMC Bioinformatics.

[79] Suyama M, Torrents D, Bork P (2006). PAL2NAL: robust conversion of protein sequence alignments into the corresponding codon alignments. Nucleic Acids Res..

[80] Langmead B, Salzberg SL (2012). Fast gapped-read alignment with Bowtie 2. Nat. Methods.

[81] Sun J, Nishiyama T, Shimizu K, Kadota K (2013). TCC: an R package for comparing tag count data with robust normalization strategies. BMC Bioinformatics.

[82] Love MI, Huber W, Anders S (2014). Moderated estimation of fold change and dispersion for RNA-seq data with DESeq2. Genome Biol..

[83] Laemmli UK (1970). Cleavage of structural proteins during the assembly of the head of bacteriophage T4. Nature.

[84] Bailey TL, Johnson J, Grant CE, Noble WS (2015). The MEME Suite. Nucleic Acids Res..

