# Supplementary material for: Integrative transcriptome analysis discloses the molecular basis of a heterogeneous fungal phytopathogen complex, Rhizoctonia solani AG-1 subgroups
Source: Sci Rep. 2019 Dec 23;9:19626. doi: 10.1038/s41598-019-55734-2 (PMC6928066; doi:10.1038/s41598-019-55734-2)
Supplement: Supplementary file 16 — Supplementary Figures [file 41598_2019_55734_MOESM16_ESM.pdf]

Integrative transcriptome analysis discloses the molecular basis of a heterogeneous fungal phytopathogen complex, *Rhizoctonia solani* AG-1 subgroups

Naoki Yamamoto[1, 2†], Yanran Wang[1, 2†], Runmao Lin[1, 2†a], Yueyang Liang[1, 3], Yao Liu[1, 2], Jun Zhu[1, 3], Lingxia Wang[1, 3], Shiquan Wang[1, 3], Huainian Liu[1, 3], Qiming Deng[1, 3], Shuangcheng Li[1, 3], Ping Li[1, 3] and Aiping Zheng[1, 2*]

[1] Rice Research Institute, Sichuan Agricultural University, Chengdu 611130, China

[2] Key Laboratory of Sichuan Crop Major Diseases, Sichuan Agricultural University, Chengdu 611130, China

[3] Key Laboratory of Southwest Crop Gene Resource and Genetic Improvement of Ministry of Education, Sichuan Agricultural University, Ya'an 625014, China

[*] Corresponding authors: Aiping Zheng (apzh0602@gmail.com)

[†] Equally contributed

[a] Present address: Institute of Vegetables and Flowers, Chinese Academy of Agricultural Sciences, Beijing 100081, China

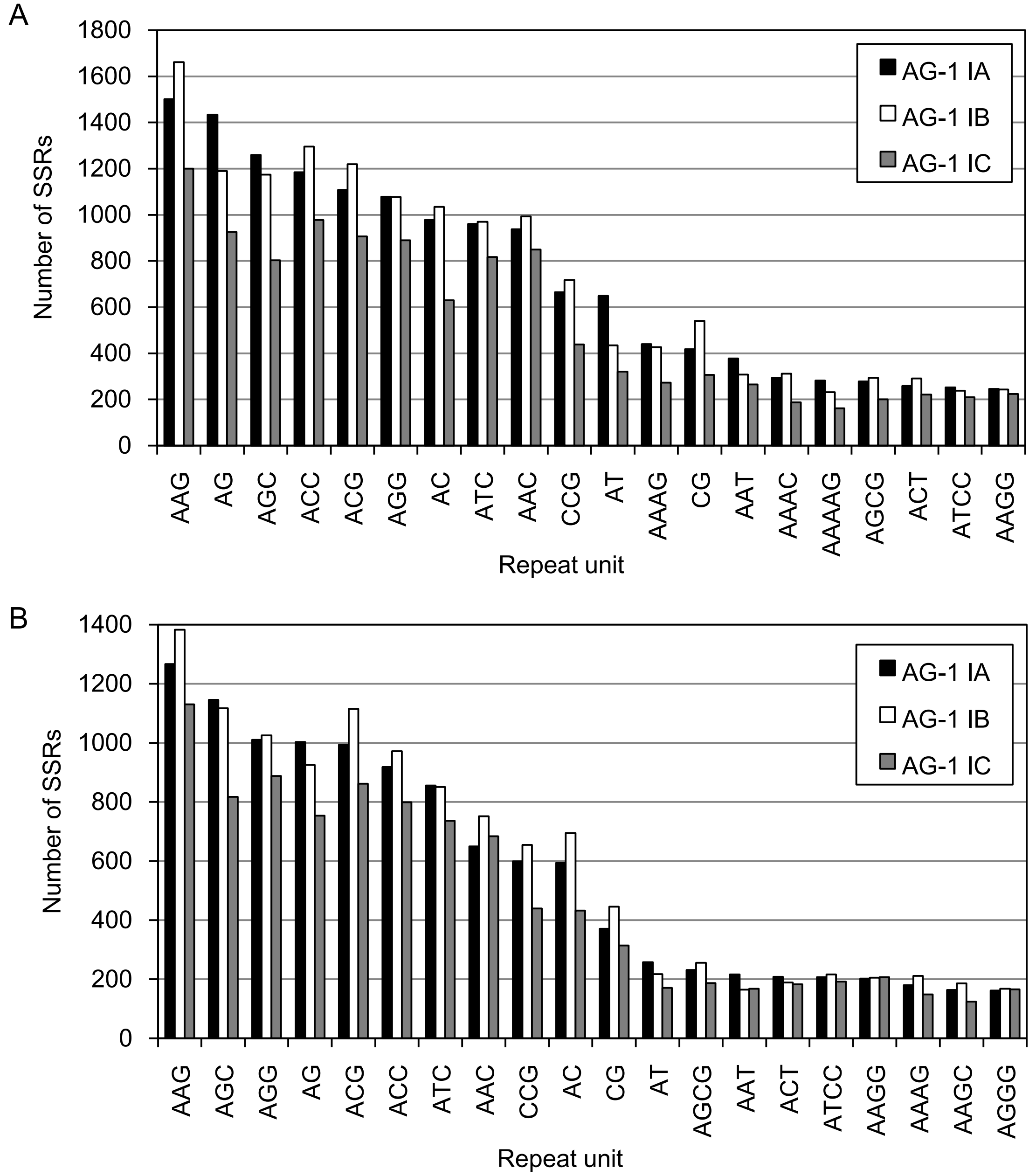

Figure S1. Frequencies of SSR repeat units on the transcriptome contigs. The top 20 of repeat units were shown. The horizontal axis represents sequence repeat units, and the vertical axis represents number of transcript SSRs comprised of each repeat unit. A: all of the SSRs, B: SSRs on coding sequences.

```
IA-TC_DN397_c0_g1_i1    CACCGCACGCCGTGATGATCGTGGGTATGGC------------GGATACAGGGAGGACCGATATG
IB-TC_DN8984_c0_g1_i1   CACTGCCCGTCGCGATGATCGCGGTTATAGC---------GGTGGATACAGGGAGGACCGATATG
IC-TC_DN9366_c0_g4_i1   TACTGCGCGCCGTGATGATCGTGGTTATAGCGGTGGTGGTGGTGGATACAGGGAAGACCGATATG

IA-TC_DN9324_c0_g2_i1   TCCGCCGGATTCGGCCAACAGCAGCAG---------------CAACAGCCTGGGCAGACGGGCAG
IB-TC_DN10912_c1_g2_i2  TCTGCCGGATTTGGCCAGCAGCAGCAGCAGCAACAACAACAACAGCAACCTGGACAAACTACCAG
IC-TC_DN8479_c0_g2_i1   TCTGCCGGATTCGGCCAACAGCAG---------------CAACAACAACCTGGGCAGACCACCAG

IA-TC_DN9712_c0_g1_i1   TTTCAATTCTCCCCTCTGCGCCTGCTGTCATAACTCATATATAGCCGTCGTCCAGGATGTCCTCT
IB-TC_DN5599_c0_g1_i3   TTTC----CTCACCTCTGCGCCTGCTGTCATATCTC----ATAGCCGCCGTCTAGGATGTCTTGT
IC-TC_DN9395_c0_g1_i1   TTT-----CCCACCTCTGCGCCCGCTGTCATATCTC--ATATAGCTGCCGTCTAGGATGTCTTGT

IA-TC_DN10228_c0_g2_i1  AGAGCATCGTACCATCCGTCTGCGCAGCAGCAACAGCAACAGTACCAAGAGAGGGCACCTTCGCC
IB-TC_DN10070_c0_g1_i1  AGAGCATCGTACCATCCGTCTGCGCAGCAAC------------ACCAAGAGAGAGCACCATCTCC
IC-TC_DN8036_c0_g2_i1   AGGGCGTCGTACCATCCATCCGCGCAACAAC------------ACCAAGAGAGGGCACCATCGCC

IA-TC_DN10336_c0_g1_i1  GTGTCCCTTCGTTGCCGTCTACTTCTTCTTCCTCTTCTTCCTGTGCTCCGAGAGGTCTGCCTCCC
IB-TC_DN8339_c0_g2_i2   GTGTTCCTTCGTTGCCGTCGACTTCTTCTTCTTCTTC------AGCCCCGAGGGGTCTCCCACCA
IC-TC_DN12374_c0_g1_i1  GTGTTCCTTCACTACCGTCTACCTCTTC---------------AGCTCCCAGAGGTCTCCCACCA

IA-TC_DN10353_c0_g1_i1  CTCTGGACTCGAACCTAAATTTCTCTCTCCCCTCCCTATCTCTCTCTCTCTCTTGGTACATTGCA
IB-TC_DN6937_c0_g1_i1   CTCTGGACTCGAATTTAAA-------------------------TCTCTCTCTGGGTACATTACA
IC-TC_DN9968_c0_g1_i1   CTCTGGACTCGAATCTAAA---------------------------TCTCTCTGGGTACATCGCA

IA-TC_DN10705_c0_g2_i1  TCTCCCACTTTCTTCCTCGACGGAATTCTGGTC---------GTGGCGGTGGTGGAAGCAGCGAT
IB-TC_DN7382_c0_g1_i1   TCCCGCACTTCCTTTCTCGGCGTAATTCTGGTCGTGGTGGTGGTGGTGGTGGTGGAAGTAGTGAT
IC-TC_DN6622_c0_g3_i1   TCTCCCACCTTCTTCCTCGGCGTAACTCTGGTC---------GTGGCGGCGGCGGAAGCAGCGAT

IA-TC_DN10833_c0_g1_i1  CCGACCAACTGG------------------------------------------CAACACCAACC
IB-TC_DN9782_c0_g2_i1   CCAACCAACTGGGCTCAGCACCAGCACCAACACCAACACCAACACCAACACCAACACCAACAGCC
IC-TC_DN5579_c1_g1_i1   CCGACCAACTGGGC------CCAACACCAACACCAACACCAGCACCAGCACCAGCACCAACAACC

IA-TC_DN11095_c0_g1_i1  CCACAGCCCTATGGCCCGCCACAGCCGCAGCCGCAGCCGCAGCCTCAGCCAACTCCATACCCTCA
IB-TC_DN10716_c0_g4_i5  CCGCAGCCCTATGGGCCACCC------------------CAGCCCCAGTCGACTCCGTATCCCCA
IC-TC_DN9493_c1_g18_i1  CCGCAGCCCTATGGACCACCC------------------CAGGCTCAGCCAACACCGTACCCTCA

IA-TC_DN11150_c0_g1_i1  AGATGGTCAGGGGGATTTGGGGTTCGAAGGAGAAGGAAAAAGAAAAGGAAAAGCCCAAGGAGCAG
IB-TC_DN10588_c0_g2_i1  AGATGGTTAGGGGAATTTGGGGCTCCAAGGAGAAG------GAAAAGGAAAAGCCGAAAGAACAA
IC-TC_DN11749_c0_g1_i1  AAATGGTCAGGGGGATTTGGGGCTCAAAAGAGAAG------------GAAAGGCCAAAGGAACAA
```

Figure S2. Probable polymorphic SSRs on IA-TCs, IB-TCs, and IC-TCs. SSRs were underlined.

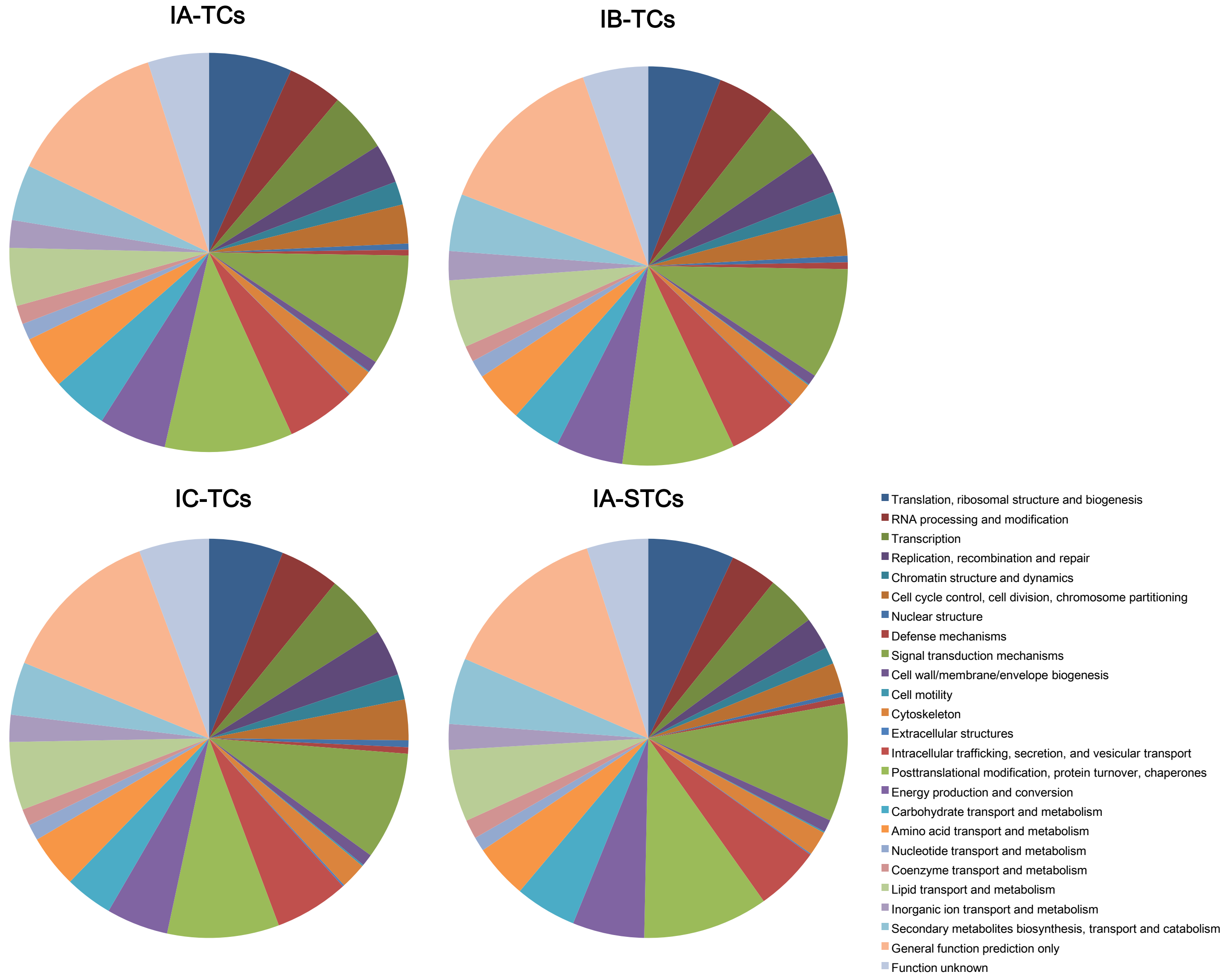

Figure S3. KOG classifications of the transcriptome contigs. The pie charts represent distributions of number of contigs annotated by KOGs.

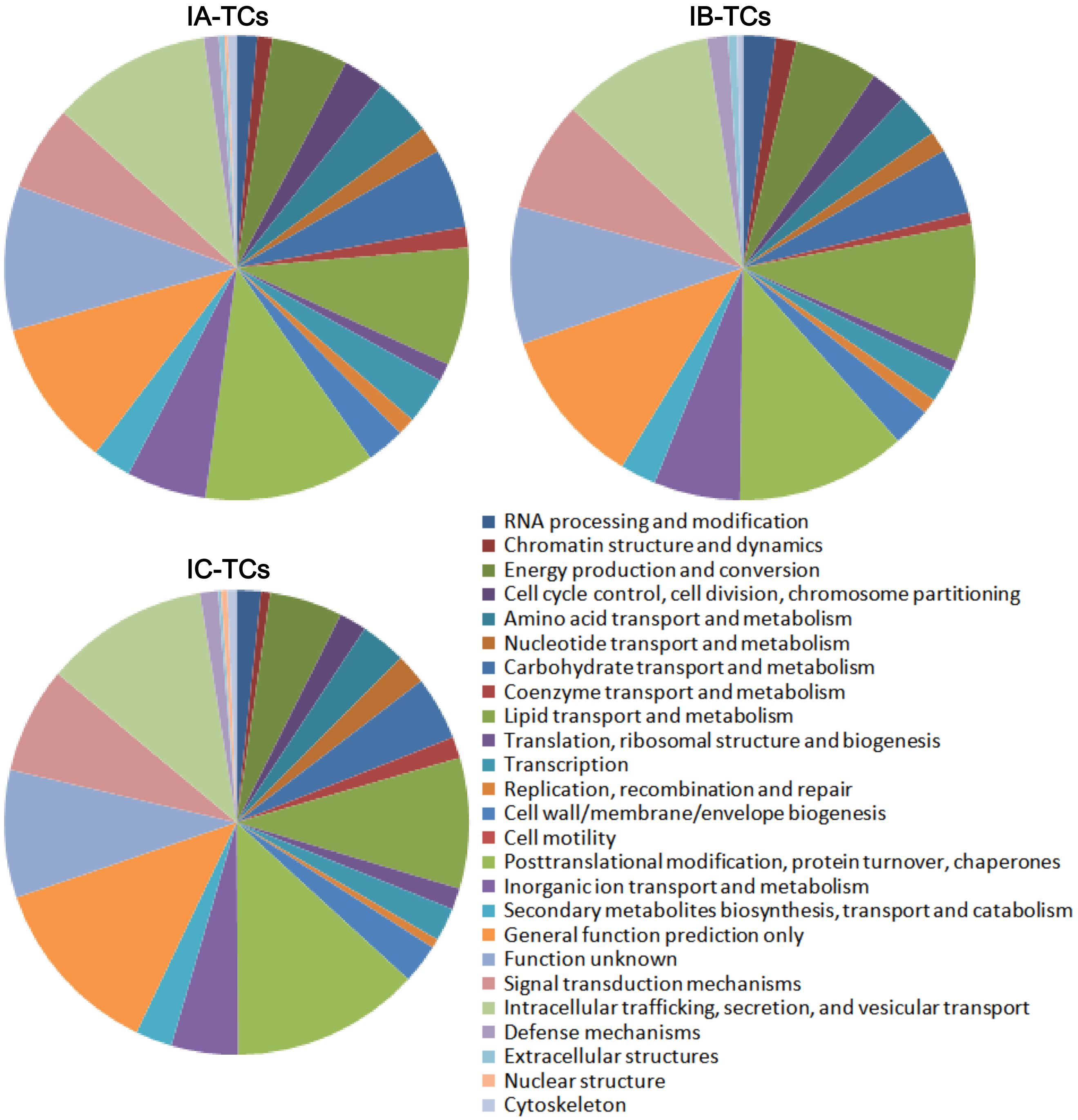

Figure S4. KOG classification of the predicted secretome proteins. The pie charts represent distributions of number of secreted gene contigs annotated by KOGs.

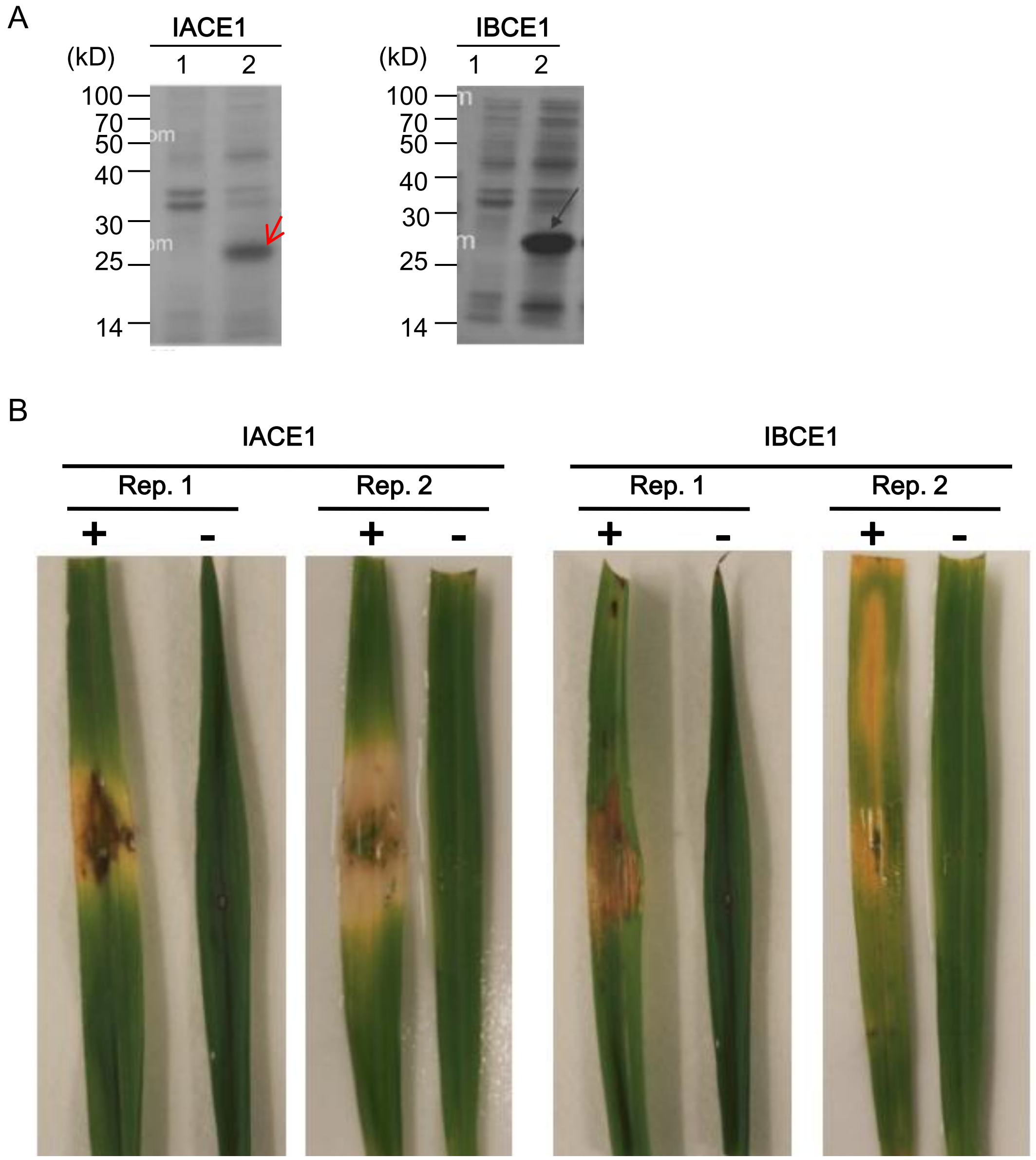

Figure S5. Bioassays for three small secreted protein genes. A: functional expression of a candidate effector IACE1 and IBCE1 in *E. coli*. Lane 1: not induced by IPTG, lane 2: induced by IPTG. Red arrows indicate recombinant proteins. B: Leaf phenotypes affected by infiltration of recombinant proteins IACE1 and IBCE1. "+" and "-" indicates presence and absence of recombinant protein. Results of two replications were shown. C: functional expression of a candidate effector IACE2 in *E. coli*. Lane 1: not induced by IPTG, lane 2: induced by IPTG. The red arrow indicates recombinant proteins. D: Leaf phenotypes affected by infiltration of recombinant proteins IACE2. "+" and "-" indicates presence and absence of recombinant protein. E: functional expression of a candidate effector IACE3 in *E. coli*. Lane 1: not induced by IPTG, lane 2: induced by IPTG. The red arrow indicates recombinant proteins. F: Leaf phenotypes affected by infiltration of recombinant proteins IACE3. "+" and "-" indicates presence and absence of recombinant protein.

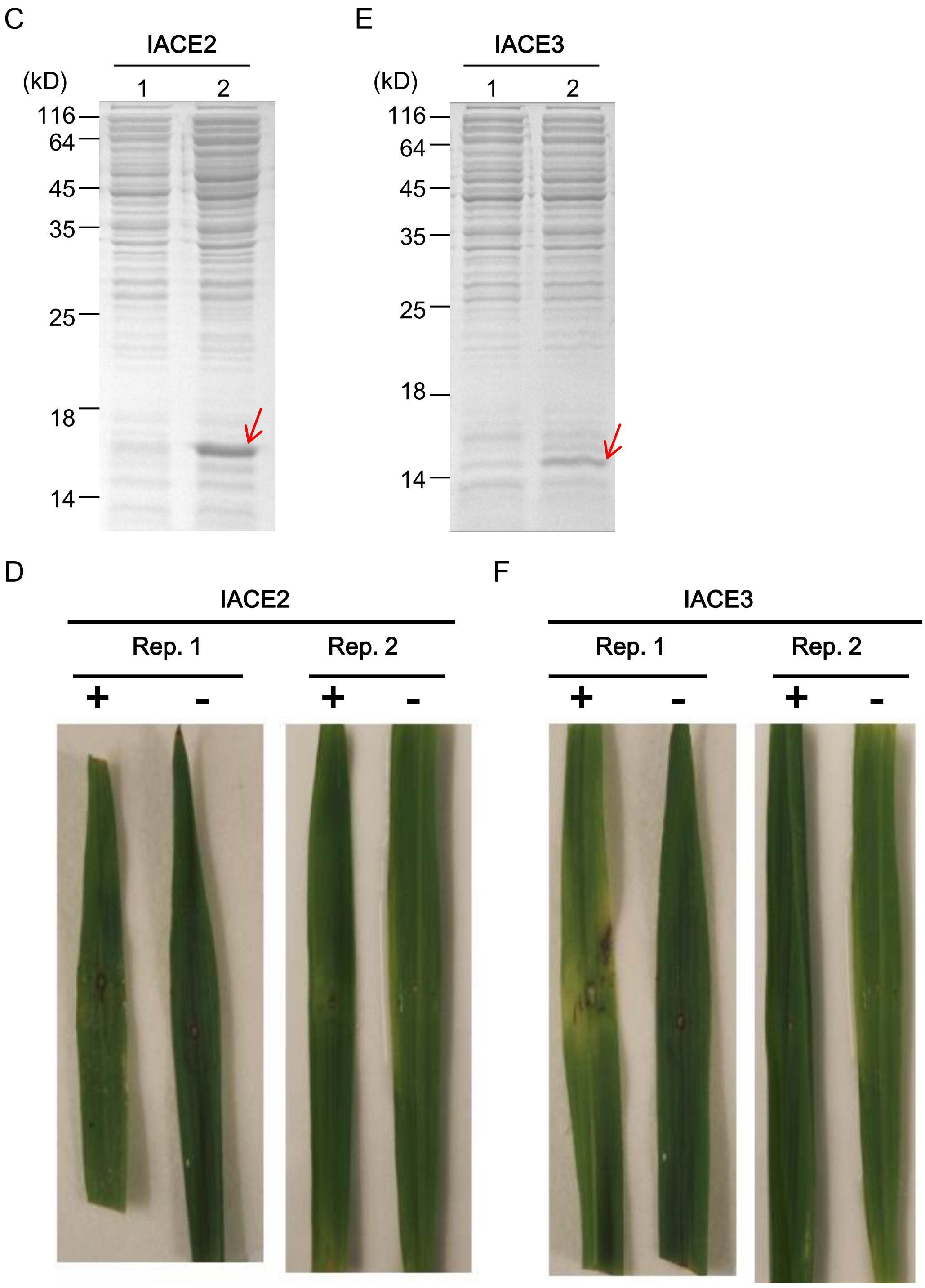

Figure S5. Bioassays for three small secreted protein genes. A: functional expression of a candidate effector IACE1 and IBCE1 in *E. coli*. Lane 1: not induced by IPTG, lane 2: induced by IPTG. Red arrows indicate recombinant proteins. B: Leaf phenotypes affected by infiltration of recombinant proteins IACE1 and IBCE1. "+" and "-" indicates presence and absence of recombinant protein. Results of two replications were shown. C: functional expression of a candidate effector IACE2 in *E. coli*. Lane 1: not induced by IPTG, lane 2: induced by IPTG. The red arrow indicates recombinant proteins. D: Leaf phenotypes affected by infiltration of recombinant proteins IACE2. "+" and "-" indicates presence and absence of recombinant protein. E: functional expression of a candidate effector IACE3 in *E. coli*. Lane 1: not induced by IPTG, lane 2: induced by IPTG. The red arrow indicates recombinant proteins. F: Leaf phenotypes affected by infiltration of recombinant proteins IACE3. "+" and "-" indicates presence and absence of recombinant protein.

```
IACE1 MLLSAVALLACSALGFASPLQRRAAPDNTVLIRSESDYCLILPRIAGATV 50
IBCE1 MLLSALALLACSALGFASPLQSRAAPDNTVFIRSESDYCLILPRTAGATV 50
ICCE1 MLFSAVALLACSAIGLASPLQPRAAPDNTVLIRSETDFCMILPRTPGATV 50
      **:**:*******:*:***** ********:****:*:*:**** .****

IACE1 GESETPGGMQAFCSPSAYTDPSQGQLPGNFWRSVTLERGSGGSGQQYVQL 100
IBCE1 GESETPGGMQAFCSPSARSNPSQGVLPGNFWRSVTLASGTGASGQPYVQL 100
ICCE1 GESETPGGMQAFCSPSARTDSSQGQLPGNFWRSVTLERGTGSSGQPYIQL 100
      ***************** ::.*** ***********  *:*.*** *:**

IACE1 TGCINLSSQLNPSDGGGQYDSSGGDGGRGNPEGSVCEGYNHYVELLEPGS 150
IBCE1 TGCINVFSQLSPGDGGGQYDSSGGDGGRGNPEGSVCEGYNHYVELLEPGS 150
ICCE1 TGCINVFSQLNPGDGGGQYDSSGGDGGRGNPEGSVCEGYNHYVELLEPGA 150
      *****: ***.*.************************************:

IACE1 GRACIRCCQDTNDCPLSMDTSGCPAVIPGNYDGC 184
IBCE1 NRACLRCCQDYNDCPLSMDTSGCPAVIPGNYNGC 184
ICCE1 NRACIRCCQDTNDCPLTMDTSGCPAVIPGNYQ-C 183
      .***:***** *****:**************: *
```

Figure S6. A protein sequence alignment of orthologous effector candidates in the three AG-1 strains. Arrows indicate Cys residues. Predicted singnal peptides were underlined. The deduced amino acid sequences were aligned by the ClustalW2.1 software (Larkin *et al*. 2007).

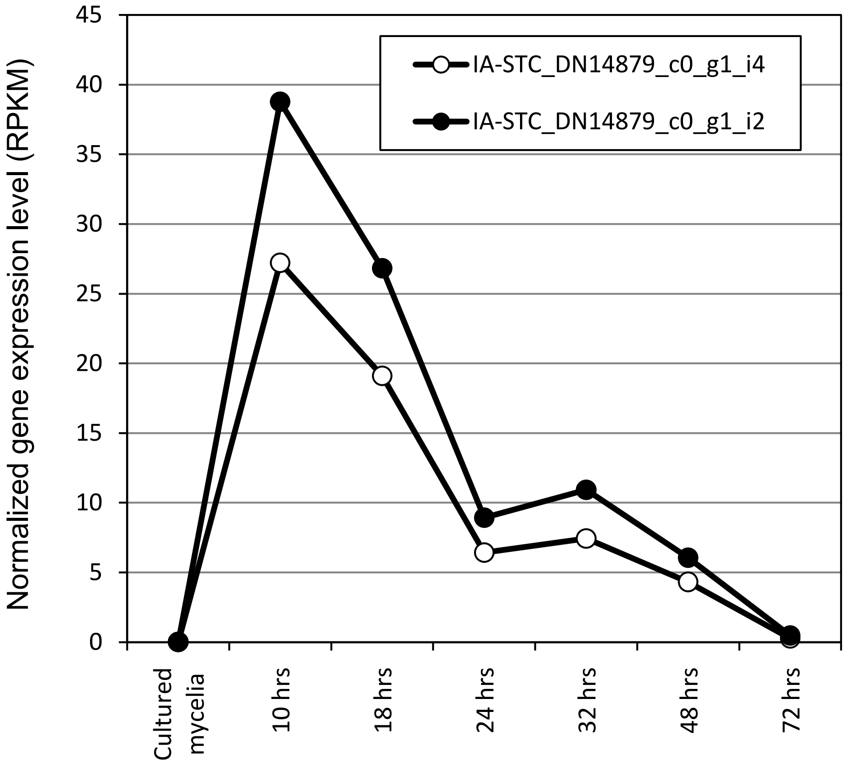

Figure S7. Gene expression patterns of the major facilitator family protein contigs. The horizontal axis represents mycelial samples: cultured mycelia (not inoculated), 10, 18, 24, 32, 48, and 72 hours after inoculation onto rice. The vertical axis gene expression levels.

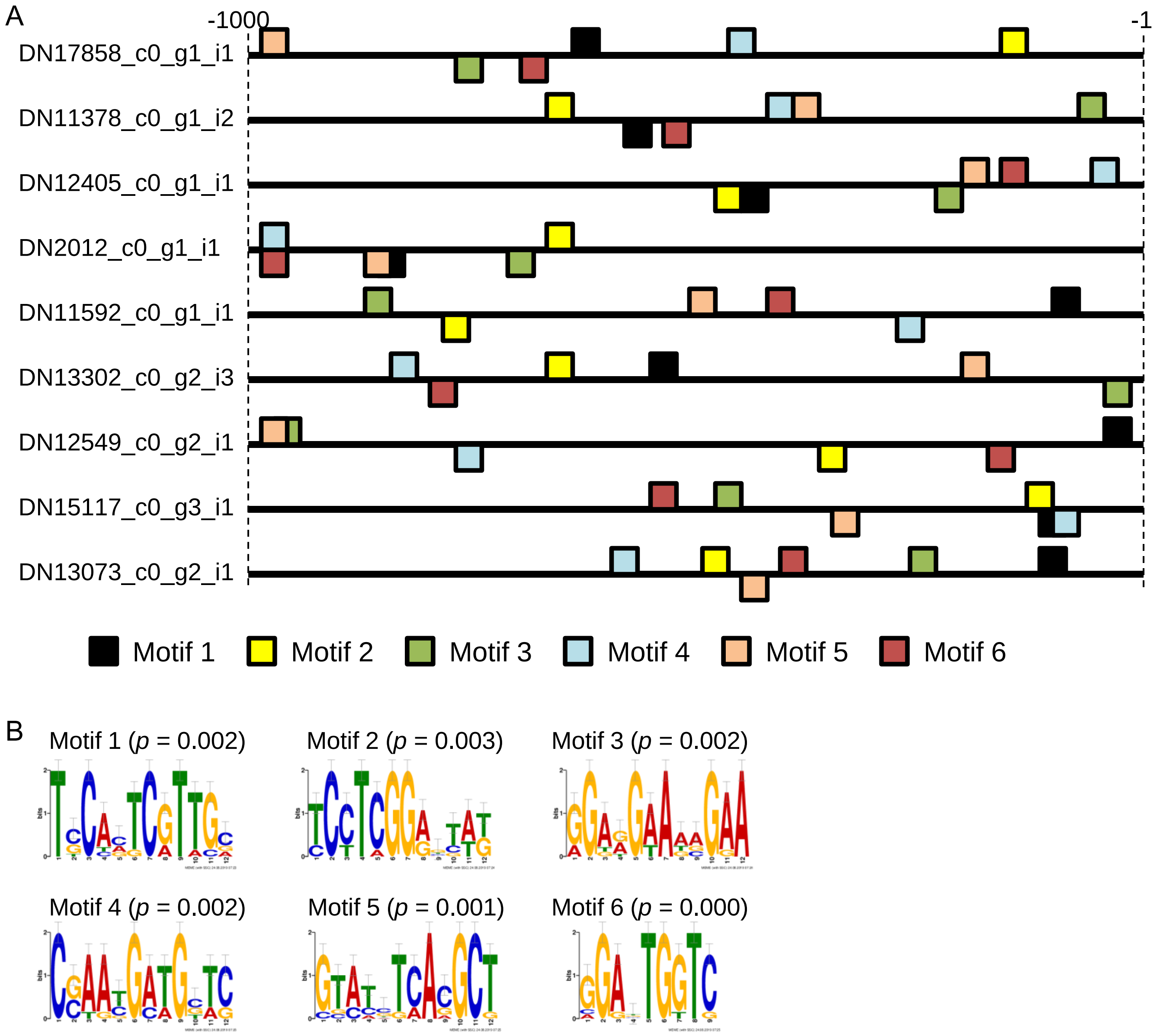

Figure S8. Cis-motif candidates on *AA9*. (A) Position of cis-motifs in 1 kb upstream of coding sequences. (B) Predicted cis-motifs. Parentheses represent *p* value in Fisher's exact test.

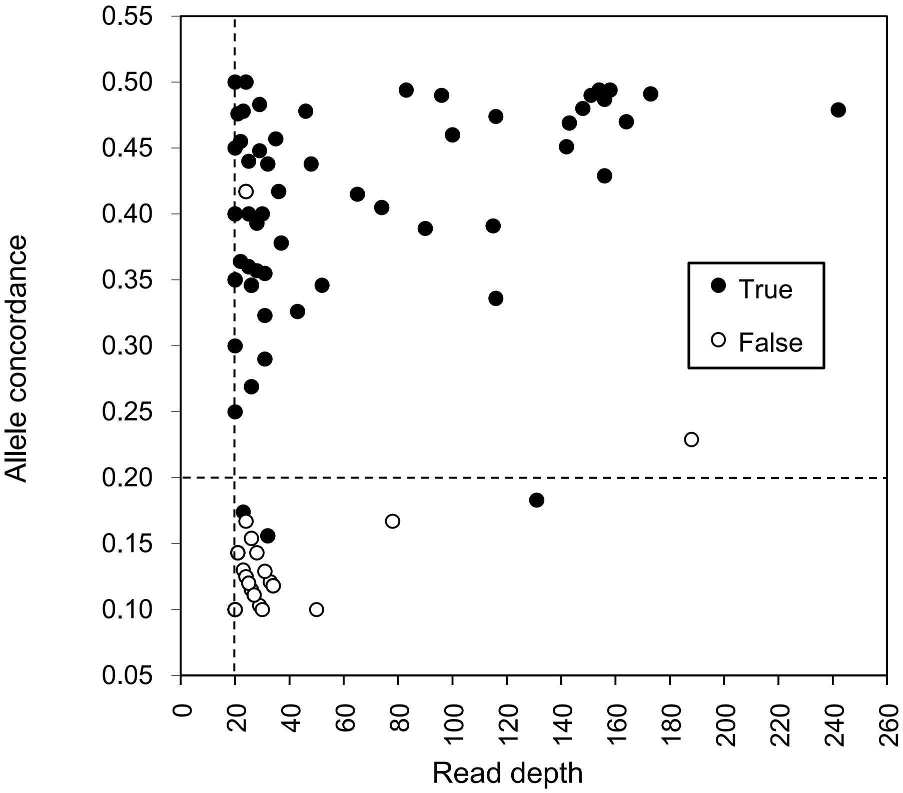

Figure S9. Cross-check of potential multinucleate SNP sites on IA-TCs by comparison to probable multinucleate SNP sites on the AG-1 IA draft genome. A hundred sites were analyzed. The horizontal axis and the vertical axis represent read depth of transcritpome reads and allele concordance of transciptome alleles, respectively.
True: validated as true, false: could not be validated.

Reference for Supplementary Figures

Larkin, M. A. *et al.* Clustal W and Clustal X version 2.0. *Bioinformatics* **23**, 2947-2948 (2007)
